# In silico APC/C substrate discovery reveals cell cycle-dependent degradation of UHRF1 and other chromatin regulators

**DOI:** 10.1371/journal.pbio.3000975

**Published:** 2020-12-11

**Authors:** Jennifer L. Franks, Raquel C. Martinez-Chacin, Xianxi Wang, Rochelle L. Tiedemann, Thomas Bonacci, Rajarshi Choudhury, Derek L. Bolhuis, Taylor P. Enrico, Ryan D. Mouery, Jeffrey S. Damrauer, Feng Yan, Joseph S. Harrison, M. Ben Major, Katherine A. Hoadley, Aussie Suzuki, Scott B. Rothbart, Nicholas G. Brown, Michael J. Emanuele

**Affiliations:** 1 Department of Pharmacology, The University of North Carolina at Chapel Hill, Chapel Hill, North Carolina, United States of America; 2 Lineberger Comprehensive Cancer Center, The University of North Carolina at Chapel Hill, Chapel Hill, North Carolina, United States of America; 3 Center for Epigenetics, Van Andel Research Institute, Grand Rapids, Michigan, United States of America; 4 Department of Genetics, The University of North Carolina at Chapel Hill, Chapel Hill, North Carolina, United States of America; 5 Department of Chemistry, University of the Pacific, Stockton, California, United States of America; 6 Department of Cell Biology and Physiology, Department of Otolaryngology, Washington University in St. Louis, St. Louis, Missouri, United States of America; 7 McArdle Laboratory for Cancer Research, Department of Oncology, University of Wisconsin, Madison, Wisconsin, United States of America; UCLA, UNITED STATES

## Abstract

The anaphase-promoting complex/cyclosome (APC/C) is an E3 ubiquitin ligase and critical regulator of cell cycle progression. Despite its vital role, it has remained challenging to globally map APC/C substrates. By combining orthogonal features of known substrates, we predicted APC/C substrates in silico. This analysis identified many known substrates and suggested numerous candidates. Unexpectedly, chromatin regulatory proteins are enriched among putative substrates, and we show experimentally that several chromatin proteins bind APC/C, oscillate during the cell cycle, and are degraded following APC/C activation, consistent with being direct APC/C substrates. Additional analysis revealed detailed mechanisms of ubiquitylation for UHRF1, a key chromatin regulator involved in histone ubiquitylation and DNA methylation maintenance. Disrupting UHRF1 degradation at mitotic exit accelerates G1-phase cell cycle progression and perturbs global DNA methylation patterning in the genome. We conclude that APC/C coordinates crosstalk between cell cycle and chromatin regulatory proteins. This has potential consequences in normal cell physiology, where the chromatin environment changes depending on proliferative state, as well as in disease.

## Abstract 2

This study shows that the cell cycle E3 ubiquitin ligase APC/C is a regulator of several chromatin regulatory proteins, including the multivalent epigenetic reader and writer UHRF1. Perturbing UHRF1 ubiquitylation and degradation alters cell cycle and DNA methylation patterning, pointing to a key role for cell cycle degradation in shaping chromatin environments.

## Introduction

Regulated protein degradation is central to cell and organismal physiology and plays a particularly important role in proliferation. In eukaryotes, protein degradation is controlled largely by the ubiquitin (Ub) system. E3 Ub ligases provide substrate specificity and facilitate the transfer of Ub onto substrates. The formation of poly-Ub chains on substrates provides a signal that often targets substrates to the proteasome for degradation [1].

The anaphase-promoting complex/cyclosome (APC/C) is a 1.2-MDa, multi-subunit E3 ligase and essential cell cycle regulator. APC/C utilizes 2 coactivators, Cdc20 and Cdh1, which directly bind substrates, recruiting them to the E3 complex [2]. APC/C^Cdc20^ becomes active in mid-mitosis and promotes the metaphase to anaphase transition. APC/C^Cdh1^ becomes active in late mitosis and remains active until the end of G1, during which time it prevents S-phase entry [3]. Thus, APC/C^Cdc20^ and APC/C^Cdh1^ play opposing roles, the former promoting cell cycle progression in mitosis and the latter inhibiting cell cycle progression in G1.

In addition to its role in normal cell cycles, APC/C dysfunction has been implicated in disease. Cdh1 is a haploinsufficient tumor suppressor in mice and cooperates with the retinoblastoma protein to restrain proliferation [4–8]. Several oncogenic kinase cascades impinge on Cdh1 function, further supporting a role for APC/C^Cdh1^ in tumor suppression [9–11]. In addition, the APC/C subunit Cdc27 is mutated in cancer and associated with aneuploidy [12]. APC/C is also linked to inherited disorders that give a range of disease phenotypes, including microcephaly, cancer predisposition, and skeletal abnormalities [13,14].

Cdh1 and Cdc20 bind substrates through short, linear sequence motifs termed degrons. The most well-defined APC/C degron motifs are the KEN-box and D-box [15,16]. In addition, the binding of Cdc20 and Cdh1 to APC/C promotes a conformational change in the E3 that stimulates ligase activity [17]. This results in substrate poly-ubiquitylation by its 2 cognate E2 enzymes. UBE2C/UbcH10 deposits the first Ub monomers onto substrates and forms short Ub chains, whereas UBE2S elongates poly-Ub chains [18–21].

Most known APC/C substrates are linked to cell cycle processes, including mitotic progression, spindle function, and DNA replication. The paramount importance of APC/C in cell cycle and non-cell cycle processes, and its dysfunction in disease, highlights the importance of systematically defining substrates, whose regulation (or dysregulation) will likely contribute to proliferation and disease phenotypes. Nevertheless, barriers exist to the identification of APC/C substrates, as well as most other E3s. E3-substrate interactions are dynamic, and binding often triggers substrate proteolysis. Additionally, the abundance of most substrates is low, and for APC/C, most targets are cell cycle regulated. Furthermore, since APC/C is a massive complex with many substrates, the relative binding stoichiometry to each individual substrate is low. Finally, degron sequences are short and occur vastly across proteomes, making it difficult to predict substrates.

We developed a simple in silico approach to identify potential APC/C targets. We took advantage of common features among known substrates, namely, their transcriptional regulation during cell cycle and the presence of a degron motif. These features were super-imposed onto the human proteome, enriching for known substrates and suggesting previously undescribed targets.

This analysis revealed a role for APC/C in chromatin biology. We validate several substrates involved in chromatin dynamics, highlighting a previously underappreciated role for APC/C in chromatin regulation. We further define the mechanisms of ubiquitylation for ubiquitin-like with PHD and RING finger domains 1 (UHRF1), a multivalent chromatin-binding protein and itself an E3 ligase that can ubiquitylate histone H3 [22–25]. UHRF1 plays an important role in DNA methylation and has been implicated in other DNA-templated processes, including DNA repair [26–28]. Additionally, UHRF1 is suggested to be an oncogene, whose expression correlates with high tumor grade and poor prognosis [29–31].

Altogether, these results reveal a role for APC/C-dependent UHRF1 degradation in cell cycle progression and shaping the DNA methylation landscape. More broadly, our data suggest that cell cycle–regulated protein degradation helps organize the epigenetic landscape during proliferation. This suggests a potential mechanistic link contributing to changes in the chromatin landscape observed between proliferating and non-proliferating cells [32,33]. We predict that altering APC/C function could promote changes in the histone and DNA modification landscape and that these effects could contribute to the biochemical and phenotypic features of diseases, including cancer and neurological disorders.

## Results

### Identification of APC/C substrates

To identify human APC/C substrates, we first performed FLAG immunoprecipitations (IP) from asynchronous HEK-293T cells expressing amino-terminal-tagged FLAG-Cdh1 or an empty vector and analyzed precipitated proteins by mass spectrometry (S2 Data). Several APC/C complex components and known substrates, including Rrm2, Kif11, Claspin, and cyclin A, were enriched in Cdh1 pulldowns. Compared with a previously established dataset [34], we identified 15 out of 53 known substrates. However, hundreds of proteins were enriched over controls and many known substrates scored weakly. For example, a single spectral count was observed for the substrate Kif22/KID [35,36]. The prevalence of non-specific interactors and other non-substrate-binding proteins confounded our ability to prioritize these data to identify new substrates.

**Fig 1:**
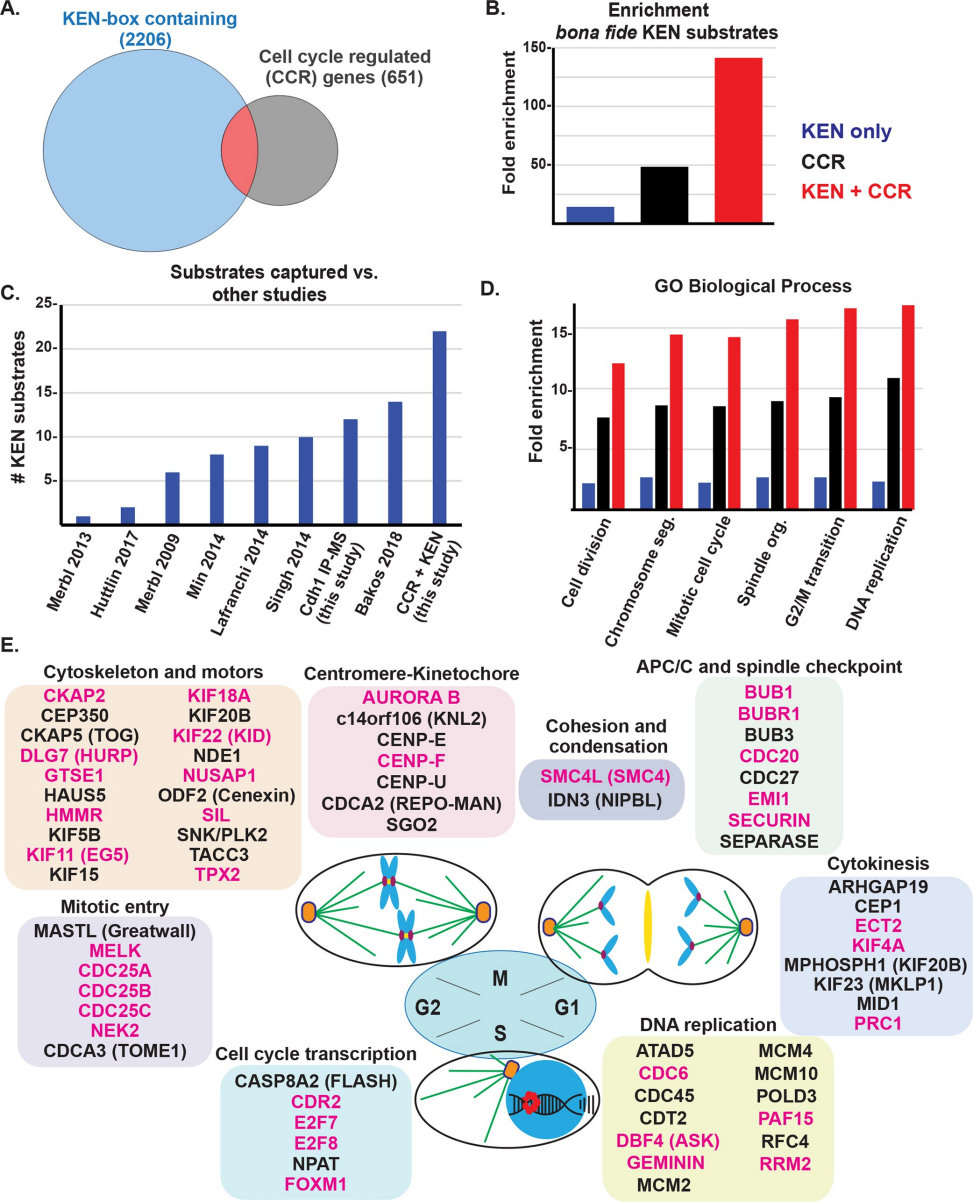
In silico analysis reveals a high confidence set of APC/C substrates involved in mitosis. (A) KEN-box containing human proteins were identified and cross-referenced against a set of 651 genes whose expression is cell cycle regulated based on multiple, independent studies. This revealed a set of 145 KEN-box containing proteins whose mRNA expression is cell cycle regulated. (B) Analysis of the enrichment of bona fide KEN-dependent substrates among these 3 datasets (blue, KEN box only set (2,206); black, cell cycle regulated mRNAs (651); red, the overlapping set of 145 proteins compared against a curated set of bona fide, KEN-dependent APC/C substrates [16]). Enrichment was calculated based on the expected number of substrates, which would be captured by chance based on the size of the dataset. (S1 Data) (C) Analysis of putative substrates recovered in the indicated studies. (S1 Data) (D) GO analysis for indicated studies (blue, KEN box only set (2,206); black, cell cycle–regulated mRNAs (651); red, the overlapping set of 145 proteins). (S1 Data) (E) The set of 145 putative substrates was manually curated and analyzed for roles in various aspects of cell cycle progression. Seventy proteins, involved in cell cycle activities, are shown. The ones labeled in magenta signify that there is evidence in the literature of their regulation by APC/C. (Note that AURORA B, a mitotic kinase that phosphorylates histone H3, is listed here and in Fig 2A). APC/C, anaphase-promoting complex/cyclosome; GO, gene ontology.

We considered computationally identifying substrates based on features common among substrates. APC/C binds substrates most often through D- and KEN-box degron motifs. The minimal D-box motif (R-x-x-L) is present in most human proteins and insufficient as a prediction tool. The KEN-motif is found in approximately 10% of human proteins (2,206; S3 Data), and several D-box-regulated substrates also contain a KEN-motif, including Securin and Cdc6 [37,38]. In addition, the gene expression of most APC/C substrates oscillates during the cell cycle [39]. We cross-referenced the KEN-motif containing proteins against a set of 651 proteins whose mRNAs scored in at least 2 cell cycle mRNA profiling studies [40–43]. Overlapping the 2,206 KEN-motif containing proteins with 651 transcriptionally controlled genes produced a set of 145 proteins, which represent known and putative APC/C substrates (Fig 1A and S3 Data).

We compared our in silico analysis with 2 previously curated datasets, 1 containing 53 known APC/C targets [34] and a second containing 33 specifically KEN-dependent APC/C substrates [16]. When compared with these lists of 53 and 33 substrates, our dataset captured 26 and 22 of them, respectively, the latter representing an enrichment of more than 140-fold, compared with what would be expected by chance (Fig 1B). We compared both our Cdh1 IP/MS dataset and in silico analysis to several other studies that identified APC/C substrates, interactors, proteins degraded at mitotic exit, or proteins ubiquitylated in mitosis (S4 Data) [34,44–49]. Our in silico analysis identified the most KEN-dependent substrates relative to these studies (Fig 1C and S4 Data). When compared to the set of 53 substrates, which includes both D- and KEN-box-dependent substrates, our dataset captured 26 out of 53 known substrates, despite not focusing on D-box substrates. Combining the in silico predictions with our Cdh1-pulldown proteomics data, we captured 31 out of 53 substrates.

Among the 145 computationally identified known and potential substrates, gene ontology (GO) analysis showed a strong enrichment for processes linked to various aspects of cell division (Fig 1D). Whereas the analysis of cell cycle genes expectedly enriched for GO terms related to cell division (Fig 1D), these same terms were more significantly enriched when the analysis was restricted to those cell cycle genes that encode proteins containing a KEN-motif. Manual curation demonstrated that nearly half of the proteins we identified (70 of 145) have well-established roles in cell cycle. These were subclassified into the following subcategories: cytoskeleton and motors, centromere-kinetochore, APC/C and spindle checkpoint, cytokinesis, mitotic entry, cell cycle transcription, cohesion and condensation, and DNA replication (Fig 1E). Among these 70 proteins, 50% have literature evidence for regulation by APC/C, highlighting our enrichment for APC/C substrates (Fig 1E; shown in magenta). All 145 proteins, their known function, subcategory, KEN-box sequence motif with flanking sequence, aliases, and citations describing regulation by APC/C are detailed in S3 Data.

### Regulated degradation of chromatin factors

**Fig 2:**
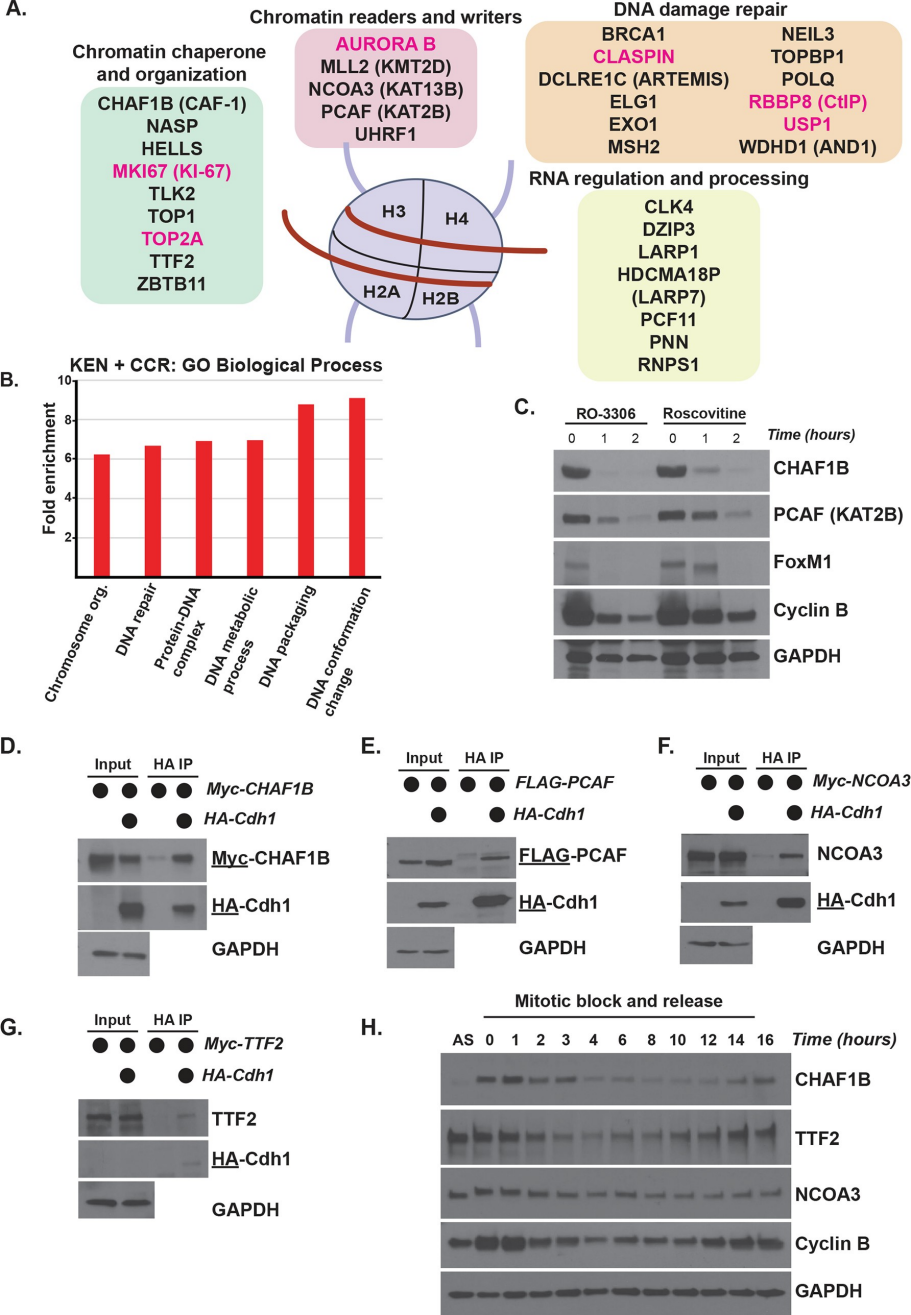
Putative APC/C substrates are enriched for roles in chromatin regulation. (A) The set of 145 known and putative APC/C substrates is enriched for proteins involved in various chromatin-related process. This includes chromatin readers and writers, chaperones, RNA regulation and processing, DNA damage repair, and others. (Note that AURORA B, a mitotic kinase that phosphorylates histone H3, is listed here and in Fig 1E) (B) GO analysis of the overlapping KEN-box containing cell cycle–regulated transcripts. This set is enriched for the indicated biological process, including DNA metabolism, protein-DNA complex assembly, DNA packaging, and DNA conformation. (S1 Data) (C) APC/C activation assay to monitor substrate degradation. Following synchronization in mitosis, cells were washed 1 time and treated with CDK inhibitors to remove inhibitory phosphorylation marks that hinder the formation of APC/C^Cdh1^ needed for the M/G1 phase transition. Protein degradation was monitored by immunoblot. CHAF1B and PCAF are putative APC/C substrates, and FoxM1 and Cyclin B are known targets. Data representative of *n* = 3 experiments. (D) coIP of HA-Cdh1 with Myc-CHAF1B in transiently transfected HEK-293T cells treated with proteasome inhibitors prior to harvesting. The underline indicates which protein or tag was blotted for in a particular panel (here and below). Input equal to 1% of IP, here and below. Data representative of *n* = 2 experiments. (E) coIP of HA-Cdh1 with FLAG-PCAF in transiently transfected 293T cells treated with proteasome inhibitors prior to harvesting. Data representative of *n* = 2 experiments. (F) coIP of HA-Cdh1 with Myc-NCOA3 in transiently transfected 293T cells treated with proteasome inhibitors prior to harvesting. Data representative of *n* = 3 experiments. (G) coIP of HA-Cdh1 with Myc-TTF2 in transiently transfected 293T cells treated with proteasome inhibitors prior to harvesting. Data representative of *n* = 4 experiments. (H) Mitotic shake-off of synchronized U2OS cells collected after release at the indicated timepoints. Immunoblotting for select endogenous proteins that are putative APC/C substrates or the positive control cyclin B. Data representative of *n* = 3 experiments. APC/C, anaphase-promoting complex/cyclosome; coIP, co-immunoprecipitation; GO, gene ontology.

Unexpectedly, our dataset revealed several proteins involved in chromatin regulation (Fig 2A) and an enrichment for GO processes related to chromatin (Fig 2B). The dataset includes readers and writers of histone posttranslational modifications, including the lysine acetyltransferases, PCAF/KAT2B and NCOA3/KAT13B, the lysine methyl-transferase MLL2/KMT2D, the chromatin reader and histone Ub ligase UHRF1, and the mitotic histone H3 kinase Aurora B (Figs 2A and 1E). We identified proteins involved in chromatin assembly and structure, including CHAF1B, a component of the CAF-1 nucleosome assembly complex; TTF2, a Swi2/Snf2 family member and DNA-dependent ATPase; KI-67, which prevents chromosome aggregation in mitosis and regulates histone posttranslational modifications; and proteins associated with cohesion and condensation such as SMC4 and NIPBL (Fig 1E). We also identified proteins involved in DNA damage repair.

To validate potential substrates, we developed an in vivo APC/C activation assay that is amenable to analysis of endogenous or exogenously expressed proteins and which is similar to approaches described elsewhere [50]. U2OS cells were synchronized in mitosis with the microtubule poison nocodazole. After harvesting cells by mitotic shake-off, CDK1 was inactivated with either the CDK1-specific inhibitor RO-3306 or pan-CDK inhibitor Roscovitine, driving cells out of mitosis and triggering APC/C activation and destruction of substrates, including FoxM1, NUSAP1, and cyclin B (Fig 2C and S1 Fig) [51].

Using a combination of exogenous expression and endogenous protein analysis, we examined the levels of chromatin-related proteins not previously shown to be APC/C substrates. Using this assay, we detected a decrease in the levels of several writers of histone modifications, including UHRF1, PCAF, TTF2, and NCOA3 (Fig 2C and S1A and S1B Fig). We observed a decrease in the levels of the chromatin assembly factors NASP and CHAF1B as well as the RNA processing proteins LARP1 and LARP7 (Fig 2C and S1A and S1B Fig). All of these have been previously identified as ubiquitylated in proteomics studies by an unknown E3 ligase [52–56].

Since the role of APC/C in chromatin regulation is not well established, we focused our attention on the potential regulation of chromatin proteins by APC/C. We determined the ability of a subset to bind Cdh1 by co-immunoprecipitation (coIP). CHAF1B, PCAF, NCOA3, and TTF2 interact with Cdh1 by coIP in 293T cells (Fig 2D–2G). Accordingly, the levels of endogenous CHAF1B, TTF2, and NCOA3 oscillate during the cell cycle in U2OS, analyzed following a nocodazole-induced block in mitosis and then released into the cell cycle (Fig 2H). PCAF levels did not decrease at mitotic exit in U2OS (S1C Fig) but do decrease at mitotic exit in HeLa cells (S1C Fig), suggesting a potentially complex regulation. Finally, we purified recombinant TTF2 and found that APC/C could trigger its ubiquitylation in vitro (S2 Fig). A table of all proteins tested in these assays and their validation is shown in S5 Data. Taken together, this analysis uncovered new APC/C substrates and a role for APC/C in controlling chromatin regulators.

### UHRF1 regulation by APC/C^Cdh1^

To further understand the function of APC/C in chromatin biology, we pursued UHRF1, a key chromatin regulator that reads and writes histone modifications. UHRF1 associates with the DNA methyltransferase DNMT1 and is required for DNA methylation [26]. UHRF1 has also been implicated in replisome assembly [57,58] and its phosphorylation oscillates during the cell cycle [59].

**Fig 3:**
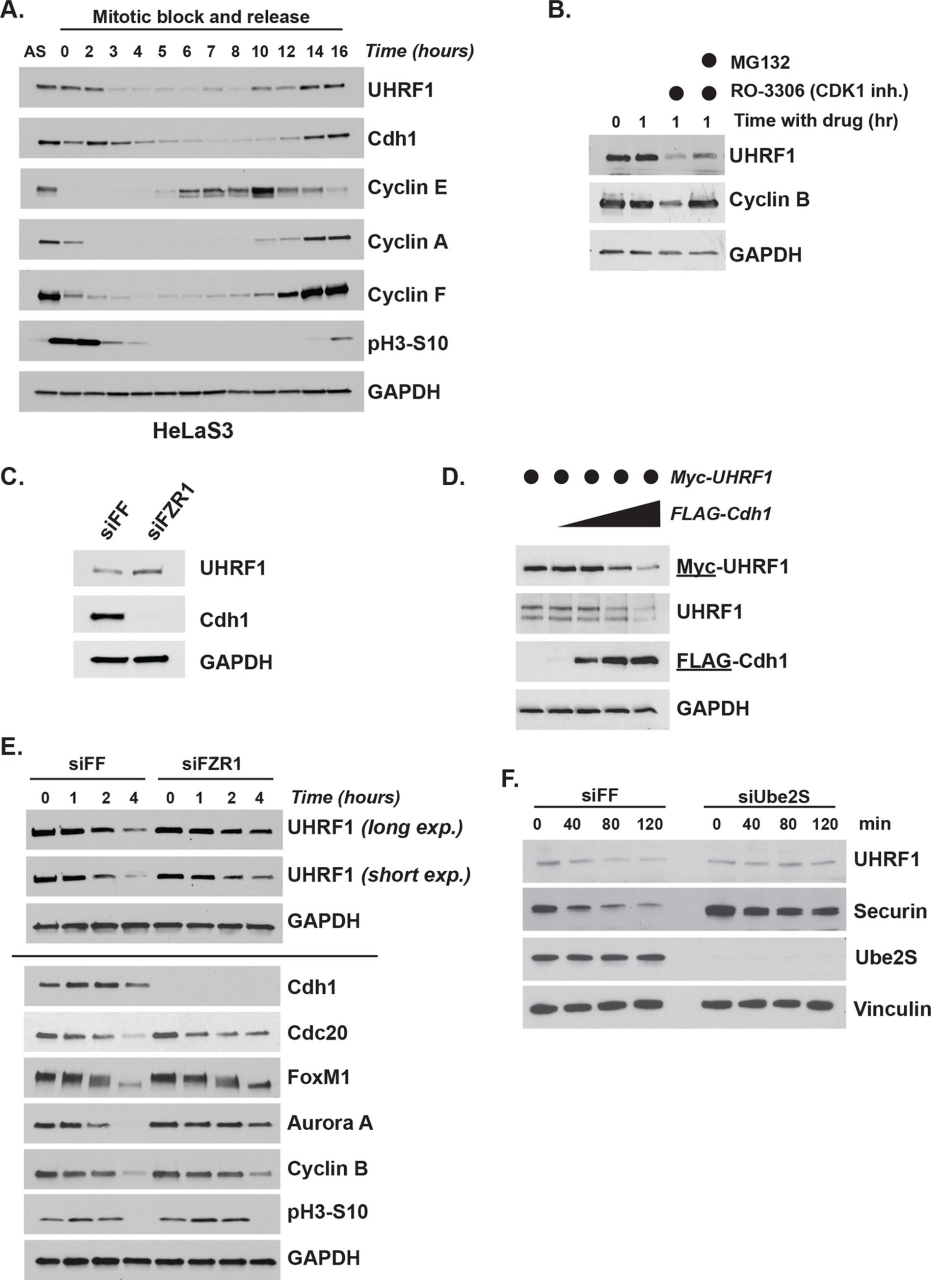
UHRF1 levels are controlled by APC/C^Cdh1^. (A) HeLa S3 cells were synchronized in mitosis and released into the cell cycle. Time points were taken at the indicated time points and analyzed by immunoblot. Data representative of *n* = 3 experiments. (B) U2OS cells were synchronized in prometaphase with 250-ng/mL nocodazole for 16 h prior to mitotic shake-off. Cells were released into fresh media containing 10-μM RO-3306 CDK inhibitor (used as described in Fig 2C) with or without addition of 20 μM of proteasomal inhibitor MG-132 and harvested 1 h later. Cyclin B is a positive control for a known APC/C substrate that is degraded at mitotic exit. Data representative of *n* = 3 experiments. (C) HCT116 cells were transfected with siRNA targeting Cdh1 (Fzr1 mRNA) or firefly luciferase as a control and harvested after 24 h for immunoblotting. Data representative of *n* = 3 experiments. (D) Myc-UHRF1 was transiently expressed in HEK-293T cells with increasing concentrations of FLAG-Cdh1 for 24 h before analysis by immunoblot. Data representative of *n* = 3 experiments. (E) HeLa S3 cells transfected with siRNA targeting FF or FZR1 for 8 h prior to synchronization in mitosis for 14 h and then released into the cell cycle. Time points were taken at the indicated time points and analyzed by immunoblot. Line indicates blots from multiple gels. Data representative of *n* = 3 experiments. (F) UHRF1 degradation assay in G1 phase-synchronized and UBE2S-depleted HeLa S3 cell extracts supplemented with ATP and Ub. Aliquots were collected at the indicated time points and analyzed by immunoblot. Data representative of *n* = 2 experiments. APC/C, anaphase-promoting complex/cyclosome; siRNA, small interfering RNA; Ub, ubiquitin; UHRF1, ubiquitin-like with PHD and RING finger domains 1.

We examined UHRF1 protein levels following a mitotic block and release. Immunoblotting for UHRF1 and other cell cycle markers showed that UHRF1 protein levels decrease during mitotic exit in HeLa S3, HeLa, and U2OS cell lines (Fig 3A and S3A and S3B Fig). In each cell line, UHRF1 levels remain low in G1 and then reaccumulate starting around G1/S-phase, based on the expression of other cell cycle markers, such as cyclin E and cyclin A, and then further increasing throughout the subsequent G2/M phase.

We performed several assays to assess whether UHRF1 is regulated by APC/C. We analyzed UHRF1 in the aforementioned in vivo APC/C activation assay. U2OS cells were arrested in mitosis and then treated with RO-3306. We observed a decrease in UHRF1 that was partially mitigated by the proteasome inhibitor, MG-132, indicating that the reduction is dependent on the proteasome (Fig 3B). In addition, transient small interfering RNA (siRNA) depletion of Cdh1 (Fzr1 mRNA transcript) augmented UHRF1 protein levels (Fig 3C). Conversely, ectopic expression of increasing concentrations of FLAG-Cdh1 led to a dose-dependent decrease in both exogenous and endogenous UHRF1 protein levels (Fig 3D). Moreover, Cdh1-depleted cells undergoing mitotic exit showed delayed endogenous UHRF1 degradation, comparable with well-established APC/C substrates (Fig 3E). Additionally, utilizing a cell-free human extract system that recapitulates APC/C substrate degradation, we observed UHRF1 levels decrease similar to Securin, a well-established APC/C substrate, and this degradation depends on the E2-conjugating enzyme UBE2S (Fig 3F). We also examined UHRF1 levels in cells that were first synchronized in G1 by a mitotic block and release and then treated with the pharmacological APC/C inhibitor proTAME for 90 min (S3C Fig). This led to an increase in endogenous UHRF1 levels. Together, these data suggest that APC/C controls UHRF1 in vivo.

### UHRF1 ubiquitylation by APC/C^Cdh1^

**Fig 4:**
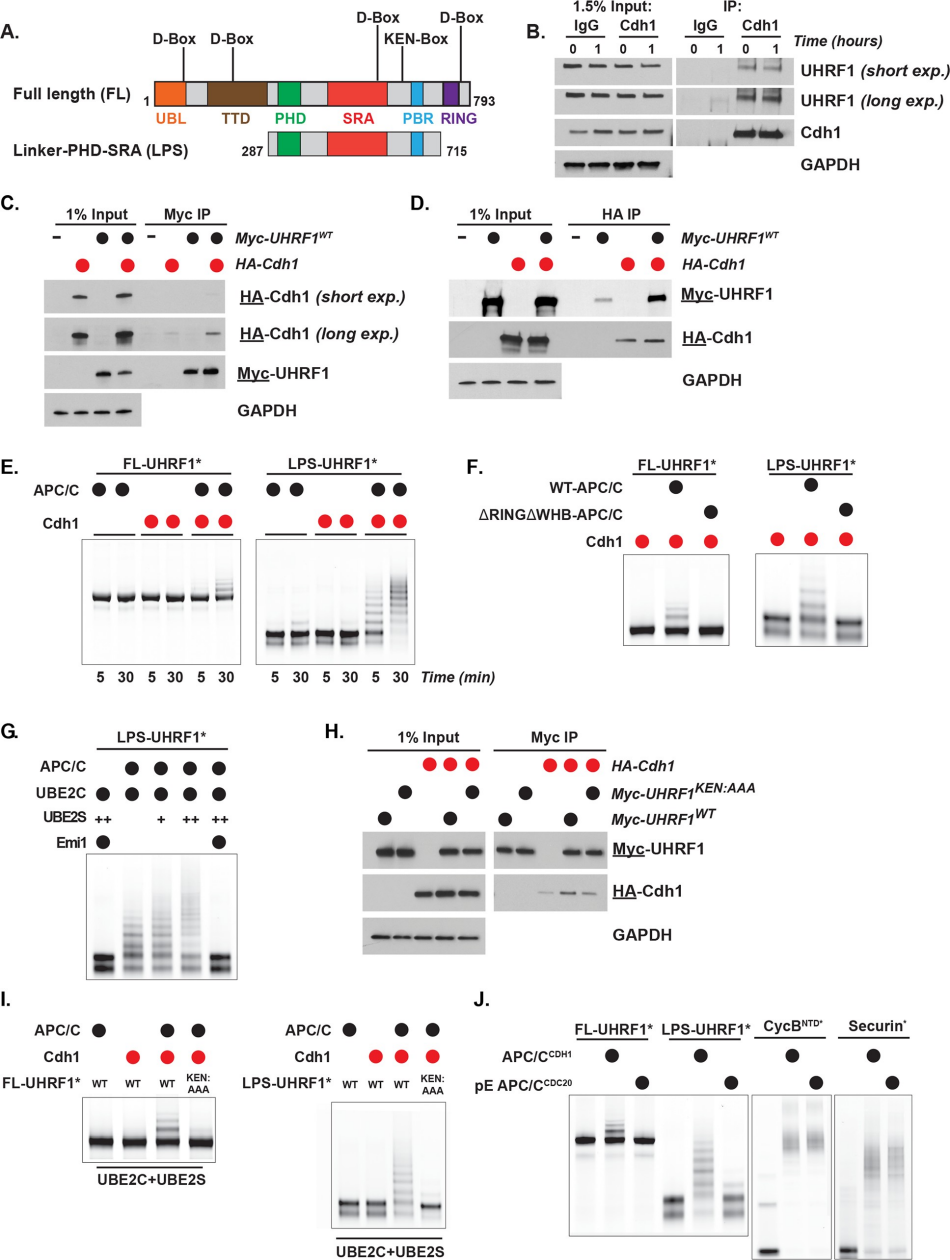
UHRF1 binding and ubiquitylation by APC/C^Cdh1^ depends on KEN degron. (A) Schematic of UHRF1 domain structure with location of KEN degron in both FL and truncated LPS UHRF1. (B) Endogenous IP of UHRF1 with Cdh1 in cells at pro-metaphase arrest and during mitotic exit. HeLa S3 cells were synchronized by nocodazole block and release as described previously. Cells were collected at 0 h and 1 h after release, flash frozen prior to α-Cdh1 IP, and analyzed by immunoblot. Data representative of *n* = 5 experiments. (C) coIP of HA-Cdh1 with Myc-UHRF1 in transiently transfected HEK-293T cells treated with proteasome inhibitors prior to harvesting and α-Myc IP. Input equal to 1% of IP, here and below. Data representative of *n* = 4 experiments. (D) coIP of Myc-UHRF1 with HA-Cdh1 in transiently transfected HEK-293T cells treated with proteasome inhibitors prior to harvesting and α-HA IP. Data representative of *n* = 3 experiments. (E) Ubiquitylation reactions with APC/C^Cdh1^, UBE2C, FL UHRF1* or LPS UHRF1*, and WT Ub. UHRF1 was detected by fluorescence scanning (* indicates fluorescently labeled protein). Data representative of *n* = 3 experiments. (F) Ubiquitylation reactions similar as in (D**)** but using 2 variants of APC/C: WT and catalytically dead APC/C^ΔRINGΔWHB^, a version of APC/C that can neither recruit nor activate its E2, UBE2C. UHRF1 was detected by fluorescence scanning. Samples were collected at 30 min. Data representative of *n* = 3 experiments. (G) Representative in vitro ubiquitylation reactions showing UBE2S-dependent chain elongation reactions of LPS UHRF1*. Titration of UBE2S: 0 μM, 0.1 μM (+), and 0.5 μM (++). The addition of Emi1 completely inhibited the reaction. UHRF1 was detected by fluorescence scanning. Samples were collected at 30 min. Data representative of *n* = 3 experiments. (H) coIP of HA-Cdh1 with Myc-UHRF1^WT^ or Myc-UHRF1^KEN:AAA^ in transiently transfected HEK-293T cells treated with proteasome inhibitors prior to harvesting and α-Myc IP. Data representative of *n* = 2 experiments. (I) Polyubiquitylation reactions of FL-UHRF1* and LPS-UHRF1* by APC/C^Cdh1^, UBE2C, and UBE2S. UHRF1 ubiquitylation by APC/C^Cdh1^ is dependent on the KEN degron motif (lane 4 in both gels). UHRF1 was detected by fluorescence scanning. Samples were collected at 30 min. Data representative of *n* = 3 experiments. (J) Dependence of UHRF1 ubiquitylation on phosphorylation state of the APC/C (referred to as pE-APC/C) and subsequent coactivator recruitment. The well-established APC/C substrates, CycB^NTD^* and Securin*, are ubiquitylated by either APC/C^Cdc20^ or APC/C^Cdh1^, whereas UHRF1 is only ubiquitylated by APC/C^Cdh1^. Reactions were run in parallel. Collections taken at 1 h (for FL and LPS UHRF1*) and 30 min (for CycB^NTD^* and Securin*). Ubiquitylated proteins were detected by fluorescence scanning. Data representative of *n* = 3 experiments. APC/C, anaphase-promoting complex/cyclosome; coIP, co-immunoprecipitation; FL, full-length; IP, immunoprecipitations; LPS, Linker, PHD, and SRA domains; Ub, ubiquitin; UHRF1, ubiquitin-like with PHD and RING finger domains 1; WT, wild-type.

UHRF1 is a multi-domain protein (Fig 4A) that exhibits multivalent binding with chromatin through histone and DNA binding domains [24,60,61]. Additionally, UHRF1 is a RING domain E3 that ubiquitylates histone H3 [22,23,25]. To determine whether UHRF1 is a direct APC/C^Cdh1^ substrate, we tested its binding to Cdh1. Endogenous Cdh1 protein interacted with endogenous UHRF1 by co-IP in mitotic synchronized cells (Fig 4B). Next, we examined binding by expressing HA-Cdh1 and Myc-UHRF1 in HEK-293T cells. Cells were treated with the proteasome inhibitor MG-132 prior to harvesting to prevent UHRF1 degradation. Myc-UHRF1 was enriched in the HA-Cdh1 pull-down, and HA-Cdh1 was enriched in the Myc-UHRF1 pull-down (Fig 4C and 4D).

Next, we purified and fluorescently labeled recombinant, bacterially expressed, full-length (FL) UHRF1 (FL-UHRF1*, where the * denotes fluorescently labeled protein). We found that FL-UHRF1* was ubiquitylated in an APC/C- and Cdh1-dependent manner using an entirely in vitro recombinant system (Fig 4E). Multiple, high molecular weight ubiquitylated forms are observed using either wild-type Ub or methylated-Ub, the latter of which cannot form poly-Ub chains. This indicates that APC/C ubiquitylates multiple lysines in UHRF1 (Fig 4E and S4A and S4B Fig).

Since UHRF1 can auto-ubiquitylate itself through its RING domain, we confirmed that its ubiquitylation is APC/C dependent. First, we purified a version of APC/C selectively missing the APC2 WHB domain and the APC11 RING domain, which are required to recruit its initiating E2 UBE2C (designated ΔRINGΔWHB) [62,63]. This version of APC/C was unable to ubiquitylate UHRF1 (Fig 4F).

Next, we purified and fluorescently labeled a truncated version of UHRF1 that contains the Linker, PHD, and SRA domains (termed (LPS)), spanning amino acids 287–715 (Fig 4A). The LPS fragment omits 3 potential APC/C D-box degron motifs, as well as the RING domain, precluding auto-ubiquitylation. A D-box motif remains in the highly structured SRA domain but is unlikely to be accessible as a degron motif [64].

Significantly, LPS-UHRF1* is more robustly ubiquitylated in an APC/C- and Cdh1-dependent manner compared to FL-UHRF1* (Fig 4E and 4F). Moreover, UHRF1 ubiquitylation is fully inhibited by the APC/C inhibitor Emi1 (Fig 4G). Ubiquitylation of UHRF1 is initiated by APC/C^Cdh1^-UBE2C, while APC/C^Cdh1^-UBE2S elongates Ub chains, indicating that UHRF1 ubiquitylation is similar to that of other substrates tested in this in vitro system (Fig 4G). We conclude that UHRF1 is a bona fide APC/C substrate.

The ubiquitylation of truncated LPS-UHRF1* (Fig 4E–4G) strongly suggests the importance of the KEN-motif, located in an unstructured region at amino acids 622–624 (Fig 4A). Alanine substitutions were introduced into the KEN sequence (UHRF1^KEN:AAA^). The KEN mutant version (Myc-UHRF1^KEN:AAA^) showed reduced, although not completely abolished, binding to HA-Cdh1 by coIP, compared with Myc-UHRF1^WT^ (Fig 4H). Additionally, the KEN mutant versions of FL-UHRF1* and LPS-UHRF1* were completely resistant to ubiquitylation by APC/C (Fig 4I). We conclude that UHRF1 ubiquitylation by APC/C^Cdh1^ is dependent on its KEN-box motif.

APC/C substrates are recruited by Cdc20 and Cdh1, and many substrates can be controlled by both coactivators. To test if UHRF1 is controlled by APC/C^Cdc20^, in addition to APC/C^Cdh1^, we used a phosphomimetic version of APC/C (termed pE-APC/C) that can utilize either Cdc20 or Cdh1, since Cdc20 cannot bind to unphosphorylated APC/C [62]. Surprisingly, unlike other, well-established APC/C substrates, including cyclin B (CycB^NTD^, amino acids 1–95) and Securin, the FL-UHRF1* and LPS-UHRF1* were ubiquitylated by APC/C^Cdh1^ but not by APC/C^Cdc20^ (Fig 4J and S4C Fig).

**Fig 5:**
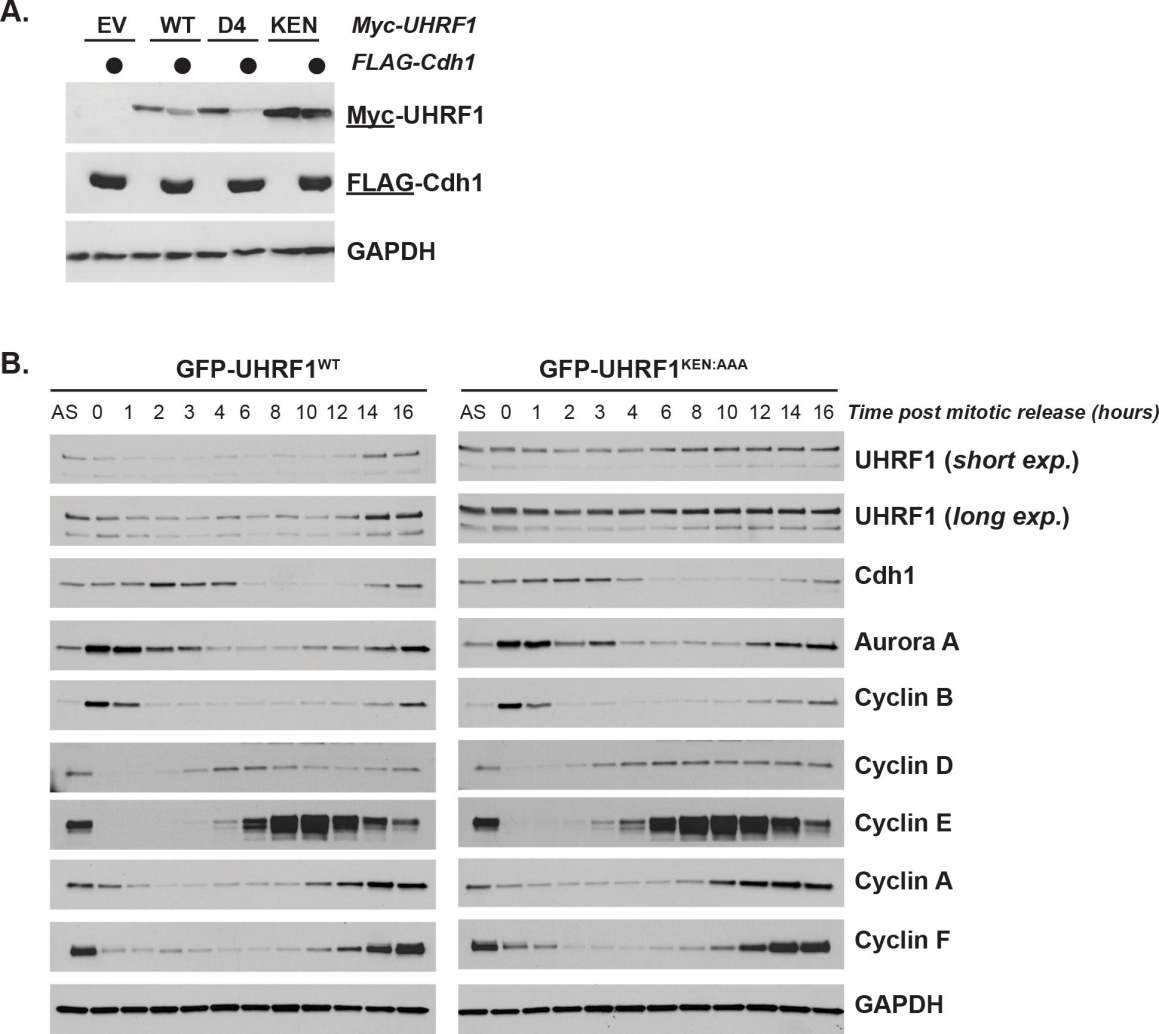
UHRF1 nondegradable mutant protein is stable at mitotic exit. (A) Myc-UHRF1^WT^ or mutant versions harboring alanine substitutions in either its KEN-box (KEN) or the fourth putative D-box motif (D4) (see Fig 4A for location of sequences) were transiently expressed in HEK-293T cells with or without FLAG-Cdh1 for 24 h before analysis by immunoblot. Data representative of *n* = 2 experiments. (B) HeLa S3 stably expressing GFP-UHRF1^WT^ or GFP-UHRF1^KEN:AAA^ were synchronized in mitosis, released into the cell cycle, and collected for immunoblot analysis at the indicated timepoints. Data representative of *n* = 3 experiments. UHRF1, ubiquitin-like with PHD and RING finger domains 1.

We transiently expressed FLAG-Cdh1 in HEK-293T cells in combination with either Myc-UHRF1^WT^ or mutant versions harboring alanine mutations in either the KEN-box (Myc-UHRF1^KEN:AAA^) or the fourth D-box motif (Myc-UHRF1^D4^). Ectopic FLAG-Cdh1 overexpression triggers the degradation of Myc-UHRF1^WT^ and Myc-UHRF1^D4^, whereas Myc-UHRF1^KEN:AAA^ is resistant to degradation (Fig 5A), further supporting the importance of the KEN-motif in UHRF1 degradation.

Next, we generated cell lines constitutively expressing GFP-tagged UHRF1^WT^ or UHRF1^KEN:AAA^ using lentiviral transduction and examined UHRF1 stability upon mitotic exit. Exogenous UHRF1 levels were only moderately overexpressed compared to endogenous levels (Fig 5B). Following synchronization with nocodazole, GFP-UHRF1^WT^ levels decrease at mitotic exit. Conversely, GFP-UHRF1^KEN:AAA^ levels remain stable through mitotic exit and G1 phase (Fig 5B). Cells expressing GFP-UHRF1^KEN:AAA^ exit mitosis normally based on immunoblotting for the APC/C substrates cyclin A, cyclin B, cyclin F, and Aurora A, which are degraded with normal kinetics (Fig 5B). Thus, the KEN-box regulates UHRF1 ubiquitylation in vitro and degradation in vivo. In addition, the mild overexpression of UHRF1 in these cells does not affect overall APC/C activity.

### UHRF1 degradation and cell cycle progression

Since many APC/C substrates are linked to proliferative control, we examined the contribution of UHRF1, and its degradation by APC/C, to cell cycle. Consistent with prior reports, UHRF1 depletion using 3 independent siRNAs increased the fraction of cells in G1 phase (S5A Fig; [65]). To further investigate the role of UHRF1 in cell cycle, we examined mitotic cells following UHRF1 depletion. We observed an approximately 3-fold increase in cells with misaligned chromosomes in metaphase and anaphase in UHRF1 depleted cells using 2 independent siRNA oligonucleotides (S5B Fig). Surprisingly, there was no statistically significant difference in the overall percentage of mitotic cells.

**Fig 6:**
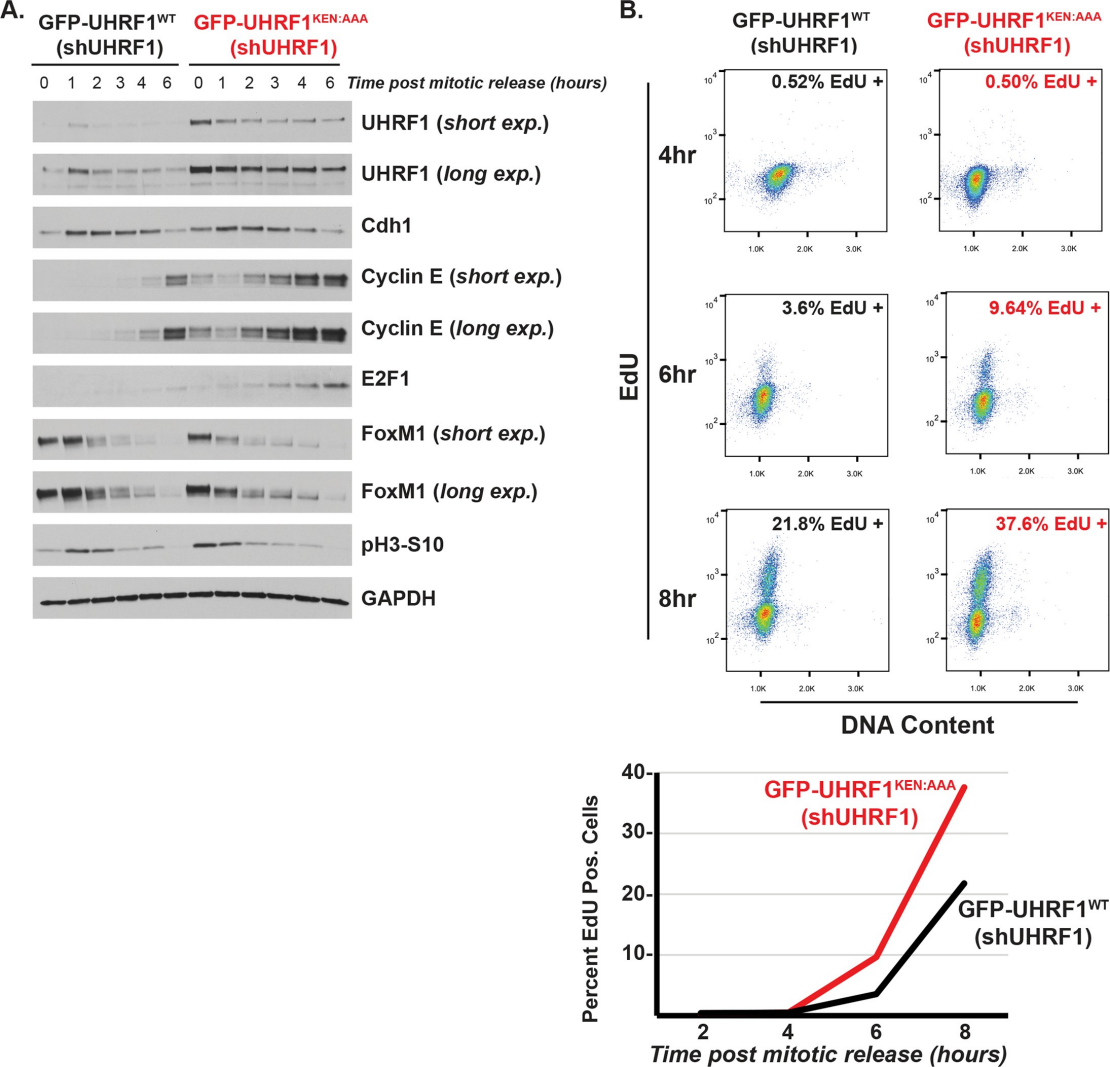
UHRF1 degradation restrains S-phase entry. (A) HeLa S3 stably expressing GFP-UHRF1^WT^ or GFP-UHRF1^KEN:AAA^ along with 3′UTR targeting shUHRF1 were synchronized in mitosis as described previously, released into the cell cycle, and collected for immunoblot analysis at the indicated time points, probing for cell cycle proteins as shown. Data representative of *n* = 3 experiments. (B) HeLa S3 stably expressing GFP-UHRF1^WT^ or GFP-UHRF1^KEN:AAA^ along with 3′UTR targeting shUHRF1 were synchronized in mitosis, released into the cell cycle, and pulsed with 10-μM EdU for 30 min prior to harvest and analysis by flow cytometry. Data representative of *n* = 3 experiments. (S1 Data). UHRF1, ubiquitin-like with PHD and RING finger domains 1.

To determine the role of UHRF1 degradation in cell cycle, we examined cell cycle markers in cells expressing UHRF1^WT^ or UHRF1^KEN:AAA^. In HeLa cells traversing the cell cycle after synchronization at G1/S, following a double thymidine block and release, we found that the GFP-UHRF1^KEN:AAA^ cells contain more of the G1/S regulator cyclin E (S6A Fig). This was also evident in cells that had been synchronized in mitosis and released into G1 (Fig 5B). This suggested that an inability to degrade UHRF1 in G1 alters cyclin E expression, a key driver of S-phase entry. UHRF1 depletion increased the percentage of G1 phase cells and expression of nondegradable mutant accelerated G1 progression. Together, these data suggested that UHRF1 might promote progression into S-phase and that a failure to degrade UHRF1 could shorten the duration of G1. To better address this possibility, we depleted endogenous UHRF1 with an shRNA targeting the UHRF1 3′UTR [66]. Cells expressing GFP-UHRF1^WT^ or GFP-UHRF1^KEN:AAA^ were synchronized in mitosis, released into the cell cycle, and analyzed by immunoblot. Increased expression of GFP-UHRF1^KEN:AAA^ is evident and consistent with its increased stability. Some residual degradation is evident in KEN-mutant expressing cells passing through G1-phase, which could be due to unrealized contributions from other degron sequences (see Discussion). Nevertheless, several markers of S-phase entry accumulate early in cells expressing GFP-UHRF1^KEN:AAA^ compared with GFP-UHRF1^WT^. Both cyclin E and the G1/S transcription factor E2F1 are elevated at early time points following release from mitosis (Fig 6A). Elevated levels of cyclin E and E2F1 are evident in asynchronous RPE1-hTRET cells and to a lesser extent in asynchronous HeLa S3 cells, where cell cycle transcription is perturbed due to HPV oncoproteins (S6B and S6C Fig).

To analyze G1 duration, cells were release from a mitotic block and pulsed with EdU prior to harvesting for flow cytometry to determine the percent of cells that were in S-phase. GFP-UHRF1^KEN:AAA^ expressing cells begin S-phase earlier than control cells (Fig 6B). Six hours after release into the cell cycle, 3.6% of control cells had entered S-phase, whereas 9.6% of GFP-UHRF1^KEN:AAA^ expressing cells had started S-phase. Thus, a failure to degrade UHRF1 accelerates G1, indicating a key role for UHRF1 destruction in determining timing between the end of mitosis and start of DNA synthesis.

### UHRF1 degradation and DNA methylation homeostasis

**Fig 7:**
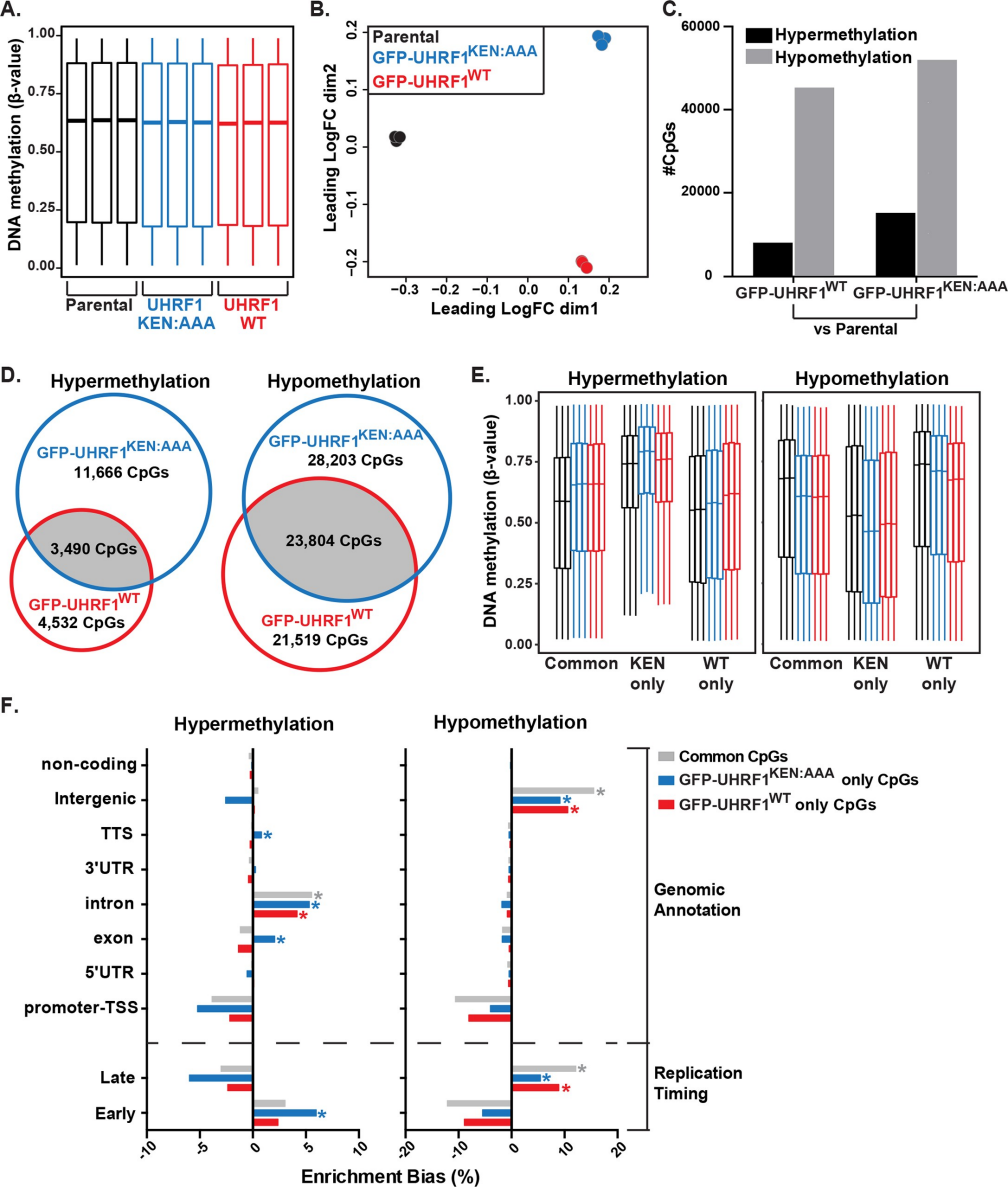
A nondegradable form of UHRF1 induces DNA hypermethylation of gene bodies and early replicating regions of the genome. (A) Global DNA methylation analysis for Parental U2OS and U2OS cells overexpressing GFP-UHRF1^WT^ or GFP-UHRF1^KEN:AAA^ with the Infinium MethylationEPIC BeadChip (Illumina) platform. Each sample group is represented in biological triplicate. All CpG probes that passed quality control analysis (*n* = 724,622 CpGs) are plotted as *β*-values population averages from 0 (fully unmethylated) to 1 (fully methylated). The midlines of each box plot represent the median DNA methylation value for all CpG probes in a sample. (S1 Data has information on accessing information for these experiments in Gene Expression Omnibus (GEO)) (B) MDS of the top 50,000 variable CpG probes among samples. (C) Number of CpG probes that were differentially hypermethylated or hypomethylated in the GFP-UHRF1^WT^ and GFP-UHRF1^KEN:AAA^ groups relative to the Parental samples adjusted *p*-value ≤ 0.05. (D) Overlap analysis of significantly hypermethylated (left) or hypomethylated (right) CpG probes between GFP-UHRF1^WT^ and GFP-UHRF1^KEN:AAA^ sample groups. (E) DNA methylation levels of significantly hypermethylated (left) or hypomethylated (right) probes from (D) that are common between GFP-UHRF1^WT^ and GFP-UHRF1^KEN:AAA^ sample groups, unique to GFP-UHRF1^KEN:AAA^ (KEN only) or unique to GFP-UHRF1^WT^ (WT only). Color code from Fig 7A applies. Outliers removed to simplify visualization. (F) Enrichment bias analysis of significantly hypermethylated (left) or hypomethylated (right) CpG probes among genomic annotations and U2OS replication timing data. *p-value ≤ 1E−300 for positive enrichment of the feature by hypergeometric testing. GEO, accession GSE137913; MDS, multidimensional scaling; UHRF1, ubiquitin-like with PHD and RING finger domains 1; WT, wild-type.

UHRF1 is required for DNA methylation maintenance [26]. To determine if stabilizing UHRF1 in G1 affects DNA methylation, we performed base-resolution DNA methylation analysis at approximately 850,000 unique human CpG loci spanning all genomic annotations and regulatory regions using the Infinium MethylationEPIC BeadChip (EPIC arrays, Illumina, Madison, Wisconsin) [67,68]. We compared parental U2OS cells and those expressing GFP-UHRF1^WT^ or GFP-UHRF1^KEN:AAA^. Considering all probes, DNA methylation changes between parental, GFP-UHRF1^WT^, and GFP-UHRF1^KEN:AAA^ were insignificant (Fig 7A). However, multidimensional scaling (MDS) of the top 50,000 variable CpG probes among all samples/replicates (agnostic of sample group) clustered experimental conditions (Fig 7B), indicating a unique and reproducible profile of methylation patterning.

We queried the GFP-UHRF1^WT^ and GFP-UHRF1^KEN:AAA^ samples for differentially methylated CpGs relative to the parental controls. Consistent with a previous report [29], expression of GFP-UHRF1^WT^ and GFP-UHRF1^KEN:AAA^ induced a comparable number of hypomethylation events (Fig 7C). Alternatively, GFP-UHRF1^KEN:AAA^ induced approximately 2-fold more hypermethylated CpGs compared with GFP-UHRF1^WT^ (Fig 7C). Analysis of differentially methylated CpG probes between GFP-UHRF1^WT^ and GFP-UHRF1^KEN:AAA^ revealed a 32% overlap in hypomethylated probes and a 17% overlap in hypermethylated probes (Fig 7D). Significantly, hypermethylated CpG probes in the GFP-UHRF1^KEN:AAA^ expressing cells were 2.5-fold more abundant compared with GFP-UHRF1^WT^, despite no significant change in hypomethylated CpG probes. Thus, the nondegradable form of UHRF1 induces site-specific DNA hypermethylation (Fig 7D).

The CpGs that were hypermethylated in GFP-UHRF1^KEN:AAA^-expressing cells started with a higher methylation level than other categories and gained methylation due to expression of nondegradable mutant (Fig 7E). Enrichment analysis of the differentially methylated CpGs revealed that gene body annotations, including exons, introns, and transcription termination sites (TTS), were positively enriched for hypermethylation in GFP-UHRF1^KEN:AAA^-expressing cells (Fig 7F, left panel). We next queried enrichment of differential methylation events in regions of early and late replication [69]. Hypermethylation events in GFP-UHRF1^KEN:AAA^, but not GFP-UHRF1^WT^, were positively enriched in early replicating regions of the genome, while hypomethylation events by both GFP-UHRF1^WT^ and GFP-UHRF1^KEN:AAA^ (alone or shared in common) were enriched in late replicating DNA (Fig 7F). The enrichment of these hypermethylated features was consistent with known DNA methylation patterns that occur across gene bodies and early replicating DNA (Fig 7E), as CpG loci in these regions typically demonstrate high levels of methylation [70,71]. Taken together, these results demonstrate that expression of nondegradable UHRF1 enhances methylation at gene-rich, early replicating regions of the genome.

## Discussion

### Identification of new E3 ligase substrates

APC/C is a core component of the cell cycle oscillator and mounting evidence points to its dysfunction in cancer and neurological disease. Here, we provide an unencumbered, annotated list of known and candidate KEN-dependent APC/C substrates. Our data highlight the importance of APC/C in various aspects of proliferative control and points to its potentially broader impact on unanticipated cellular processes, including chromatin organization.

Identifying E3 substrates remains technically challenging. Since E3-substrate interactions exhibit low stoichiometry, mapping substrates by defining interactors is difficult. In addition, Ub ligase substrates are often in low abundance. APC/C is inhibited throughout the cell cycle by myriad mechanisms [72], and the time when APC/C binds substrates coincides with when targets are being degraded and thus at their lowest abundance. This complicates many proteomics-based approaches. Alternative techniques for identifying E3 ligase substrates, including Global Protein Stability Profiling (GPS) and in vitro expression cloning, circumvent these challenges by measuring changes in substrate stability using fluorescent reporters or metabolic labeling with radioisotopes. These represent powerful tools for mapping E3 substrates [56,73]. However, both approaches are laborious and time intensive, require significant technical expertise, and depend on gene expression libraries, which are neither complete nor available to most laboratories. We bypass these challenges using a simple in silico approach based on publicly available information, which is simple, inexpensive, and easily repeated with different variables. While our approach shares some similarities with previous approaches, it improves upon those in its simplicity, expanded use of multiple cell cycle mRNA datasets, and inclusion of a degron motif in the search criteria [35,39,74]. Its success stems from the use of orthogonal filtering criteria, that is, unlinked features between mRNA and proteins. While our current approach was limited to substrates whose mRNAs are cell cycle regulated and proteins that contain a KEN-box degron, repeating this analysis with datasets capturing cell cycle protein dynamics, as they come available and more reliable, along with other known degrons, represents an interesting future approach. We predict that similar uses of unrelated properties could be leveraged for mapping targets of other enzymes such as kinases where defining substrates has proven similarly challenging. It is notable that degron sequences remain unknown for most Ub ligases, highlighting the importance of mechanistic studies in enabling systems-level discoveries.

### Involvement of APC/C in chromatin regulation

Determining the enzymes and substrates in kinase signaling cascades has been instrumental in determining proliferative controls in normal cells, their responses to stress and damage, and disease phenotypes and treatments. Relatedly, decoding Ub signaling pathways involved in proliferation is likely to provide insight into enzyme function in normal cell physiology as well as in disease.

A major finding of this work is that numerous chromatin regulators are controlled temporally during proliferation by APC/C. Impairing the degradation of one such substrate, UHRF1, altered the timing of cell cycle events and changed global patterns of DNA methylation. Since numerous chromatin regulators are controlled by APC/C, we anticipate widespread, pleiotropic effects on chromatin in cells where APC/C activity is impaired, either physiologically or pathologically.

Our observations raise the possibility that dysregulation of the cell cycle machinery, as is seen in diseases such as cancer, could alter the chromatin environment. The discovery that many chromatin regulators are mutated in cancer, a disease of uncontrolled proliferation, together with our data, imply a bidirectional relationship between the chromatin landscape and the cell cycle oscillator. Consistent with the notion that dysregulation of APC/C controlled proteins could play important roles in determining the chromatin environment in disease, the mRNA expression of our 145 known and putative substrates strongly predict breast cancer aneuploidies and copy number variations (S7 Fig). This observation is not due solely to the selection of specific breast cancer subtypes since our gene signature is elevated in multiple breast cancer subtypes. Interestingly, the expression of this signature correlates with the CIN70 signature, which was previously developed based on gene expression in chromosomally unstable cancers [75]. We observed an extraordinary correlation between the CIN70 and our 145 gene signature in breast cancer (S7 Fig). This is remarkable since our signature was generated completely independent of gene expression in cancer and was instead derived, in part, by short sequence motifs on proteins.

Multiple lines of in vitro and in vivo evidence support the regulation of UHRF1 by APC/C during the cell cycle. However, *UHRF1* mRNA expression is also regulated during the cell cycle. In fact, cell cycle–dependent transcription was an inclusion criterion for our in silico analysis since most known APC/C substrates are dynamically expressed in cycling cells. Accordingly, it remains difficult to assess the relative contributions of mRNA expression and protein degradation to overall protein expression during cell cycle. It is also unknown if other ubiquitin ligases might control UHRF1 during cell cycle, as is the case for some APC/C substrates [76,77]. The overall contributions of mRNA expression or additional E3s to the dynamics of protein expression during cell cycle are unknown for most APC/C substrates. Others have sought to address these concerns for some APC/C substrates with live cell imaging of exogenous, transiently expressed, and fluorescently tagged proteins. For UHRF1, and many other APC/C substrates, these studies represent an area of future investigation.

APC/C^Cdh1^, but not APC/C^Cdc20^, ubiquitylates UHRF1 in vitro. This ubiquitylation is dependent on a KEN-box motif in UHRF1. Interestingly, there are several other potential D-box motifs in UHRF1 that could also contribute to APC/C binding, particularly since APC/C can bind D-box and KEN-box motifs simultaneously [15,63]. This UHRF1 ubiquitylation is notable because the Cdh1-bound form of APC/C is active both G1 and quiescent cells and is critical for restraining S-phase entry. Our findings suggest that impaired UHRF1 degradation promotes a premature G1/S transition. We propose that the proper degradation of UHRF1, and other chromatin regulators, serves to integrate growth factor-dependent proliferative decisions with the chromatin regulatory environment. This regulation could help explain the complex chromatin rearrangements observed in quiescent cells, where APC/C^Cdh1^ is active [32,33,78]. Further, APC/C controls key cell cycle transcriptional regulators, including the G2/M transcription factor FoxM1 and the repressor E2F proteins, E2F7 and E2F8 [77,79]. Thus, our data point to a higher-order role regulatory role for APC/C in gene regulation, by controlling transcription factors (i.e., FoxM1), transcriptional repressors (i.e., E2F7, E2F8,), and chromatin modifiers. A few studies have also linked UHRF1 to DNA damage repair [80], and it is also possible that this role of UHRF1 contributes to altered cell cycles and the expression of cell cycle proteins in our assays.

Aberrant DNA methylation is a hallmark of cancer [81]. UHRF1 promotes DNA methylation maintenance, and too much or too little UHRF1 expression is detrimental to methylation stasis [26,29]. It is interesting to speculate that the redistribution of DNA methylation in disease could be caused, in part, by the aberrant stabilization of UHRF1, resulting from APC/C^Cdh1^ inactivation. In the future, it will be important to determine if oncogene activation acts through the APC/C to reorganize the chromatin landscape. Furthermore, determining Ub ligase substrates, like UHRF1, that might be dysregulated in pathological settings via altered degradative mechanisms could suggest therapeutic strategies to reverse their effects.

## Materials and methods

### Computational identification of putative APC/C substrates

Human proteins containing a KEN-box sequence (amino acid sequence K-E-N) were identified using the “Find a Sequence Match” feature on the Scansite web search platform (currently https://scansite4.mit.edu/4.0/#home). Proteins with cell cycle–regulated mRNA were curated from 4 independent cell cycle transcriptional studies [40–43]. The genes that scored in 2 or more of these screens were previously compiled in the supplemental data of Grant and colleagues [41]. Gene and protein name conversions were performed using the DAVID online tool (https://david.ncifcrf.gov/conversion.jsp). The overlapping set 145 proteins, which contain a KEN sequence and exhibit oscillating cell cycle–regulated mRNA expression, were identified. For all 145 proteins, we manually curated information on their alias, function, sequence flanking the KEN motif, and evidence for regulation by APC/C from various online databases and repositories, including UNIPROT, PubMed, and Genecards.

The set of 33 well-validated, KEN-containing human APC/C substrates was derived from [16]. Our own FLAG-Cdh1 IPs were compared with other APC/C substrate discovery efforts [47,48]. Singh and colleagues identified “clusters” of proteins whose levels changed at mitotic exit. For each cluster, they reported a top percentile, and for the clusters that most accurately revealed APC/C substrates [1–3], we compile their data in S4 Data in terms of which KEN-dependent substrates were identified. Their data from Cluster 1, which identified the most KEN-containing APC/C substrates, are shown in Fig 1C. Lafranchi and colleagues rank ordered proteins based on the degree of change from mitosis to G1, analyzed by proteomics. We curated their data to identify the cut-off point where the last KEN-dependent APC/C substrate was identified among their rank-ordered list. Since they provided no cut-off point, the data comparison in Fig 1C represents the best estimate of their ability to capture APC/C substrates.

### Cell culture

HeLa, HeLa S3, U2OS, HEK-293T, RPE-1, and HCT116 cells were grown in 10% fetal bovine serum (FBS) with high glucose DMEM without antibiotics. Cell culturing utilized standard laboratory practices whereby cells were grown and incubated at 37°C containing 5% CO_2_. Frozen cell stocks were stored under liquid nitrogen in 10% DMSO/90% FBS.

GFP-UHRF1^WT^ and GFP-UHRF1^KEN:AAA^ stable overexpression cells were generated by transducing HeLa S3, U2OS, and RPE-1-hTERT cell lines with pHAGE-GFP lentivirus that had been produced in HEK-293T cells. Infections were performed in the presence of 8-μg/mL polybrene for 48 h prior to antibiotic selection. Cells were selected for 5 to 7 days with 8 μg/mL (HeLa S3 and U2OS) or 10 μg/mL (RPE-1) Blasticidin. Lentiviral particles were produced by transfecting HEK-293T cells with Tet, VSVg, Gag/pol, and Rev viral packaging vectors together with the pHAGE-GFP lentiviral vectors using *Trans*IT MIRUS (cat no. MIR 2700). Viral particles were collected 48 and 72 h after transfection and stored at −80°C prior to transduction.

To generate the rescue cell lines, the U2OS and HeLa S3 stable GFP-UHRF1^WT^ and GFP-UHRF1^KEN:AAA^ expression cell lines were transduced with previously described and validated pLKO.1 lentiviral vectors encoding either shControl or 3′UTR targeting shUHRF1 [66], using 8 μg/mL polybrene to aid infection. After 48 h, cells were selected with 2-μg/mL Puromycin for 3 to 5 days. Viral particles were produced by transfecting HEK-293T cells with the pLKO.1 constructs and psPAX2 and pMD2.G packaging vectors using *Trans*IT MIRUS, collected after 48 and 72 h as mentioned previously.

Mitotic block was induced by treating 25% confluent HeLa S3 cells with 2 mM thymidine for 24 h. After washing the plates 3 to 4 times with warm media and incubating in drug-free media for 3 to 4 h, cells were treated with 100 ng/mL nocodazole for 11 h prior to harvesting by mitotic shake-off. Samples were washed 3 or 4 times with warm media, counted, and replated for indicated time points.

To synchronize cells in G1/S, HeLa S3 were plated at 20% confluency prior to addition of 2 mM thymidine. After 16 h, cells were washed 3 times with warm media and left to incubate for 8 h before the second block in 2-mM thymidine for another 16 h. Cells were washed 3 times in warm media and collected at specific time points as they progressed through the cell cycle.

To transiently inactivate the APC/C, HCT116 or U2OS cells were treated with 15 μM proTAME (Thermo Fisher, Waltham, Massachusetts, cat no. I-440-01M), a pan-APC/C inhibitor [82], for 90 min prior to harvest and immunoblotting. Cells had been released from nocodazole-induced mitotic block for 90 min in drug-free media prior to addition of drug.

### In vivo APC/C activation assay

A total of 70% to 80% confluent U2OS cells were transfected with the indicated plasmids for 24 h and then exchanged into fresh media. Alternatively, untransfected cells were used to analyze endogenous proteins. After an 8-h incubation in fresh media following transfection, cells were treated with 250-ng/mL nocodazole for 16 h. Mitotic cells were isolated by shake-off, washed once in prewarmed media, counted, and divided equally among 15-mL conical tubes. Cells in suspension were treated with DMSO, RO-3306 (10 μM), Roscovitine (10 μM), or MG-132 (20 μM) for the indicated amount of time at 37°C. Identical volumes of cells were removed from cell suspensions by pipetting, isolated by centrifugation, and frozen at −20°C prior to processing for immunoblot.

### Cell-free, G1 extract APC/C substrate degradation assay

UHRF1 degradation in G1 phase-synchronized HeLa S3 cell extracts was performed as described in [83] Briefly, confluent HeLa S3 cells were seeded to 25% confluence (3 million cells) in 15-cm plates. Next day, cells were transfected with 20 nM of control FF or 2 independent UBE2S siRNAs [18] using Lipofectamine RNAiMAX reagent (Life Technologies) according to the manufacturer’s protocol. After 8 h, 2 mM thymidine was added to the cell medium for 24 h, after which cells were washed with warm PBS once, twice with Dulbecco’s Modified Eagle Medium (DMEM), and released for 4 h before treatment with 100 ng/mL of nocodazole in DMEM for 11 h. To obtain a G1 phase population, cells were washed as described previously and released for 2 h before collection. Extract preparation was performed exactly as described [84,85]. The resulting G1 extracts were mixed in a 1:1 ratio with SB buffer and supplemented with energy mix and Ub to monitor APC/C substrate degradation. Reactions were incubated at 30°C for the indicated times, quenched with equal volume of 2× SDS sample buffer, boiled, and analyzed by SDS-PAGE and western blot.

### Molecular biology

Plasmid transfection of HEK-293T, U2OS, and HCT116 was performed with either *Trans*IT MIRUS or PolyJet (cat no. SL100688) at 1:3 or 1:4 DNA:plasmid ratio on cells with 50% to 60% confluency. After 24 h, the media was changed, and cells were expanded to larger dishes as needed. Samples were collected 24 to 48 h after siRNA transfection was performed using a 1:3 ratio of RNAi oligonucleotide to RNAiMAX (cat no. 13778–030). UHRF1 was cloned into the indicated lentiviral vectors mentioned previously using standard gateway recombination cloning. Other APC/C substrates tested for binding to Cdh1 or degradation in the APC/C activation assay were obtained from either the ORFeome collection and cloned into the indicated vectors using gateway recombination cloning or from Addgene (S6 Data) [86].

### Cell lysis and immunoblotting

Cells were lysed on ice for 20 min in phosphatase lysis buffer (50 mM NaH_2_PO_4_, 150 mM NaCl, 1% Tween-20, 5% Glycerol (pH 8.0) filtered) or NETN (20 mM Tris (pH 8.0), 100 mM NaCl, 0.5 mM EDTA, 0.5% NP40) supplemented with 10 μg/mL each of aprotonin, pepstatin A, and leupeptin; 1 mM sodium orthovanadate; 1 mM NaF; and 1-mM AEBSF (4-[2 aminoethyl] benzenesulfonyl fluoride). Following incubation on ice, cell lysates were centrifuged at (20,000 × *g*) in a benchtop microcentrifuge at 4°C for 20 min. Protein concentration was estimated by BCA assay (Thermofisher cat no. PI-23227) according to manufacturer’s protocol. Cell extracts were diluted with SDS-PAGE Gel Loading Buffer (Laemmli Buffer) prior to analysis by SDS-PAGE. Typically, 20 to 40 μg of protein were loaded on SDS gels (either BioRad 4% to 12% Bis-Tris or homemade SDS-PAGE gels) and separated at 140 to 200 V for approximately 1 h. Proteins were transferred by wet-transfer methods onto nitrocellulose membrane, typically at 100 V for 1 h or 10 to 17 V overnight at 4°C. Nitrocellulose membranes were then incubated with TBST (137 mM NaCl, 2.7 mM KCl, 25 mM Tris (pH 7.4), 0.5% Tween-20) supplemented with either 5% bovine serum albumin or non-fat dry milk for at least 1 h or overnight at 4°C. Blocked membranes were incubated overnight with primary antibodies at 4°C, washed in TBST, incubated in appropriate secondary antibodies for 1 h at room temperature, and then developed by chemiluminescence using Pierce ECL (ThermoFisher) or Clarity ECL (Bio-Rad, Hercules, California). See reagent list in the Supplement information for detailed primary and secondary antibody information.

### Immunoprecipitation

For exogenous coIP experiments, cells were lysed in NETN for 20 min on ice and then centrifuged in a benchtop centrifuge on maximum speed (20,000 × *g*) for 20 min at 4°C, prior to determining protein concentration by either Bradford or BCA assay. A master mix of 1 to 2 mg/mL protein concentration was calculated, 10% of which was retained as input while the remaining 90% was used for coIP. Prior to coIP, 50 μL of antibody-coated beads were prewashed with 1 mL of 1× PBST (0.1% Tween-20) 3 times and then preblocked with 1 mL PBS/1% BSA for 1 h at 4°C. Clarified cell lysates were also precleared by incubation with the same volume (50 μL) of empty Protein A/G agarose beads, rotating at 4°C for 1 h. After preblocking, beads were washed 3 times in lysis buffer, using low-speed centrifugation to collect beads. Buffers were removed using a small orifice, gel-loading tip to limit bead aspiration between washes. After preclearing the lysates, they were centrifuged at low speed to collect Protein A/G beads at the bottom of the tubes. Samples were carefully pipetted from the same tubes without disturbing the resin. Cell lysates were immunoprecipitated for 2 to 4 h at 4°C with 50 μL of EzView M2- or Myc-antibody beads (F2426-1ML or E6654-1ML). After coIP, beads were pelleted at low-speed centrifugation, washed 3 times with wash buffer (NETN containing additional 1% Triton-X-100, no inhibitors added), and transferred to new microfuge tubes for 1 final wash with lysis buffer to remove unbound/contaminating proteins. Wash buffer was removed from beads using small orifice, gel-loading tip as above. After removal of the final wash, beads were resuspended in 50 μL of 2× SDS-PAGE Gel Loading Buffer (Laemmli Buffer) and boiled 5 to 10 min at 95°C. Samples were removed from the beads using a 27-gauge needle to avoid bead aspiration after boiling and transferred to new microfuge tubes. Typically, 20 μL of the coIP was loaded alongside 1% of the input volume. Samples were analyzed by immunoblotting as described.

For endogenous coIP, liquid nitrogen, flash-frozen pellets previously stored at −80°C were resuspended in phosphate lysis buffer containing protease and phosphatase inhibitors (as described above) for 20 min on ice and then centrifuged in a benchtop centrifuge on maximum speed (20,000 × *g*) for 20 min at 4°C, prior to determining protein concentration by BCA assay. A master mix of 4.2 mg/mL protein concentration was calculated, 10% of which was retained as input while the remaining 90% was used for coIP. Prior to coIP, approximately 130 μL slurry of SureBeads Protein G magnetic beads/sample (Bio-Rad, #161–4023) was prewashed 3 times with 1 mL of 1× PBST (0.1% Tween-20) and 1 time with lysis buffer before incubating the beads with cell extract for 1 h, rotating at 4°C. Cell lysates were incubated overnight, rotating at 4°C, with 2-μg antibody/mg protein (8.4 μg) using either control mouse IgG (Santa Cruz, Dallas, Texas, #sc-2025) or mouse Cdh1 (clone DCS-266) (Santa Cruz, #sc-56312) antibodies. After coIP, magnetic beads were incubated with antibody-containing cell lysates for 1 to 2 h, rotating at 4°C. Then, beads were centrifuged briefly and pelleted using magnetic rack. Beads were washed by gentle pipetting 3 times with phosphate lysis buffer (without any inhibitors) and then transferred to new microfuge tubes for 1 final wash to remove unbound/contaminating proteins. After aspiration of the final wash, beads were resuspended in 50-μL 2X SDS-PAGE Gel Loading Buffer (Laemmli Buffer) and boiled for 10 min at 70°C. Samples were removed from the boiled beads with the magnetic rack and transferred to new microfuge tubes. Samples were analyzed by immunoblotting as described.

### Protein purification

Substrates for in vitro ubiquitylation assays were expressed as N-terminal GST-TEV-fusion (TTF2) or His-MBP-TEV-fusions (FL-UHRF1^WT^, LPS-UHRF1^WT^, FL-UHRF1^KEN:AAA^, LPS-UHRF1^KEN:AAA^) in BL21 (DE3) codon plus RIL cells. TTF2 was purified by glutathione-affinity chromatography, treated with TEV protease to liberate GST, and further purified by ion exchange chromatography. UHRF1 wild-type and variants were purified by amylose-affinity chromatography, treated with TEV, and followed by ion exchange chromatography. Fluorescently labeled substrates were generated by incubating 1 μM Sortase, 20× 5-carboxyfluorescein (5-FAM)-PEG-LPETGG peptide, and substrates in 10 mM HEPES (pH 8), 50 mM NaCl, and 10 mM CaCl_2_. After 2 h of incubation at 4 *°*C, reactions were stopped by removing the His_6_-tagged Sortase by nickel affinity chromatography. Then, excess 5-FAM-LPETGG was removed by size exclusion chromatography.

Expression and purification of UBA1, UBE2C, UBE2S, recombinant APC/C and pE-APC/C, Cdh1, Cdc20, Emi1, ubiquitin, and methylated ubiquitin were performed as described previously in Brown and colleagues [87–91].

### APC/C ubiquitylation assays

Qualitative assays to monitor APC/C-dependent ubiquitylation were performed as previously described [91]. In brief, reactions were mixed on ice, equilibrated to room temperature before the reactions are initiated with Ub or meUb, and quenched at the indicated time points with SDS. TTF2 ubiquitylation was monitored by mixing 100 nM APC/C, 1 μM Cdh1, 5 μM UBE2C, 5 μM UBE2S (when indicated), 1 μM UBA1, 5 μM TTF2, 5 mM Mg-ATP, and 150 μM Ub or meUb (S2 Fig). Ubiquitylation of UHRF1 wild type or its variants by APC/C were performed with 100 nM APC/C or pE-APC/C, 1 μM Cdh1 or Cdc20, 0.4 μM UBE2C, 0.4 μM UBE2S (when indicated), 1 μM UBA1, 0.4 μM UHRF1, 5 mM Mg-ATP, and Ub or meUb (Fig 4 and S4 Fig). Following SDS-PAGE, ubiquitylation products of the fluorescently labeled substrates were resolved by SDS-PAGE and imaged with the Amersham Typhoon 5 (Cytiva Life Sciences, Logan, Utah).

### Flow cytometry cell cycle analysis

HeLa S3 GFP-UHRF1^WT^ and GFP-UHRF1^KEN:AAA^ (shUHRF1) cells were synchronized in mitosis by sequential thymidine-nocodazole treatment as described above, using 2 mM thymidine and 100 ng/mL nocodazole. After release, cells were pulsed with 10 μM EdU 30 min prior to collection at specific time points. After counting the cells, 2 million cells were retained for western blotting (WB) analysis, and 1 million cells were fixed for flow cytometry. For WB, cells we pelleted and washed once with cold PBS prior to freezing at −20°C. For flow cytometry, cells were fixed in 4% formaldehyde/PBS for 15 min at room temperature. Cells were pelleted and resuspended in 1% BSA/PBS and stored overnight at 4°C. In the next day, cells were pelleted and resuspended in 1% BSA/PBS/0.5% Triton X-100 for 15 min at room temperature. Cells were pelleted, resuspended with labelling solution (100 mM ascorbic acid, 1 mM CuSO_4_, 2 μM Alexa Fluor 488 azide in PBS), and incubated for 30 min in the dark at room temperature. After addition of 1% BSA/PBS/0.5% Triton X-100, cells were pelleted and stained with 1 μg/mL DAPI in 1% BSA/PBS/0.5% Triton X-100 for 1 h in the dark at room temperature. Flow cytometry was performed on an Attune Nxt Flow Cytometer (Thermo Fisher Scientific). Channel BL1 was used for Azide 488 dye. Channel VL1 was used for DAPI dye. Following acquisition, data were analyzed using FlowJo software.

For the siRNA UHRF1 depletion experiments, asynchronous U2OS cells were transfected with 3 independent siRNAs against UHRF1 for 48 h prior to a 30 min EdU pulse (10 μM). Samples were collected for flow cytometry as described above, except for 70% ethanol fixation and 4°C overnight storage prior to staining and analysis.

### Immunofluorescence imaging

HeLa cells were plated on poly-L-lysine-coated #1.5 coverslips. Next day, cells were treated with siRNA (control siFF and siUHRF1) and RNAiMax according to manufacturer’s protocol (Invitrogen). After 48 h of siRNA treatments, cells were fixed in 3% paraformaldehyde in PHEM buffer (60 mM PIPES, 25 mM HEPES, 10 mM EGTA, 2 mM MgCl_2_ (pH 7.0)) for 15 min at 37°C. Then, cells were washed with PHEM buffer and permeabilized using 0.5% of Nonidet P-40 in PHEM buffer for 15 min at room temperature. Cells were washed and then blocked with 5% BSA in PHEM. Primary antibodies used were α-CENP-C (MBL:1:1000) as a kinetochore marker and α-tubulin (Sigma, St. Louis, Missouri: 1:500). Samples were incubated in primary antibody solution for 1 h at 37°C. All fluorescently labeled secondary antibodies (anti-mouse Alexa 488, anti-guinea pig 564) were diluted 1:200 dilution, and cells were incubated for 1 h at 37°C. DNA was counterstained with DAPI for 15 min at room temperature after washing out secondary antibodies. All samples were mounted onto glass slides in Prolong Gold antifade (Invitrogen). For image acquisition, three-dimensional stacked images were obtained sequentially at 200 nm steps along the *z* axis through the cell using MetaMorph 7.8 software (Molecular Devices, San Jose, California) and a Nikon Ti-inverted microscope equipped with the spinning disc confocal head (Yokogawa, Sugar Land, Texas), the Orca-ER cooled CCD camera (Nikon, Melville, New York), and an ×100/1.4 NA PlanApo objective (Nikon).

### Genomic DNA isolation for methylation analysis

Genomic DNA was isolated from Parental U2OS cells and U2OS cells overexpressing either GFP-UHRF1^WT^ or GFP-UHRF1^KEN:AAA^. All samples groups were processed in biological triplicates. Briefly, cells were lysed overnight at 37°C in 2 mL of TE-SDS buffer (10 mM Tris-HCl (pH 8.0), 0.1 mM EDTA, 0.5% SDS), supplemented with 100 μL of 20 mg/mL proteinase K. DNA was purified by phenol:chloroform extraction in 3 phases: [1] 100% phenol, [2] phenol:chloroform:isoamyl alcohol (25:24:1), and [3] chloroform:isoamyl alcohol (24:1). For each phase, the aqueous layer was combined with the organic layer in a 1:1 ratio. Samples were quickly shaken, allowed to sit on ice for approximately 5 min, and then separated by centrifugation at 1,693 RCF for 5 min at 4°C. The top aqueous layer was then transferred to a new tube for the next organic phase. Following extraction, DNA was precipitated with 1/10 volume 3 M sodium acetate (pH 4.8) and 2.5 volume 100% ethanol and stored overnight at −20°C. Precipitated DNA was pelleted by centrifugation at 17,090 RCF for 30 min at 4°C. The pelleted DNA was washed twice with 70% ethanol, allowed to dry for 15 min, and resuspended in TE buffer (10 mM Tris-HCl (pH 8.0), 0.1 mM EDTA). Samples were then treated with 1 mg/mL RNAse A at 37°C for 30 min and then repurified by ethanol precipitation as described above.

### Infinium methylation EPIC BeadChip (EPIC array)

Genomic DNA was quantified by High Sensitivity Qubit Fluorometric Quantification (Invitrogen), and 1.5 μg of genomic DNA was submitted to the Van Andel Institute Genomics Core for quality control analysis, bisulfite conversion, and DNA methylation quantification using the Infinium Methylation EPIC BeadChIP (Illumina) processed on an Illumina iScan system following the manufacturer’s standard protocol [67,68].

### EPIC array data processing

All analyses were conducted in the R statistical software (Version 3.6.1) (R Core Team [92]). R script for data processing and analysis is available in S1 Text.

Raw IDAT files for each sample were processed using the Bioconductor package “SeSAMe” (Version 1.2.0) for extraction of probe signal intensity values, normalization of probe signal intensity values, and calculation of *β*-values from the normalized probe signal intensity values [93–95]. The *β*-value is the measure of DNA methylation for each individual CpG probe, where a minimum value of 0 indicates a fully unmethylated CpG and a maximum value of 1 indicates a fully methylated CpG in the population. CpG probes with a detection *p*-value of >0.05 in any one sample were excluded from the analysis.

### Genomic and replication timing annotation

CpG probes were mapped to their genomic coordinate (hg38) and were then annotated to their genomic annotation relationship (promoter-TSS, exon, etc.) using HOMER (Version 4.10.3) [96].

Repli-seq data for U2OS cells used for determining CpG probe localization relative to replication timing was generated by Dr. David Gilbert’s lab (Florida State University) as part of the 4D Nucleome project (Experiment #4DNEXWNB33S2) [69]. Genomic regions were considered early-replicating if the replication timing value was >0 and late-replicating if <0. CpG probes were annotated for replication timing domains by intersecting the Repli-seq genomic coordinates with CpG probe coordinates using BEDTools (Version 2.16.2) [97].

### Identification of differentially methylated CpG probes

The Bioconductor package “limma” (Version 3.40.6) was used to determine differential methylation among sample groups and perform MDS analysis [94,95,98]. For statistical testing of significance, *β*-values were logit transformed to *M*-values: $M={log}_{2}\left(\frac{\beta }{1-\beta }\right)$. *M*-values were then used for standard limma workflow contrasts to determine differential methylation of U2OS GFP-UHRF1^WT^ or GFP-UHRF1^KEN:AAA^ overexpression to Parental U2OS cells [98,99]. CpG probes with an adjusted *p*-value of ≤0.05 were considered significant, and log fold-change of the *M*-value was used to determine hypermethylation (logFC > 0) or hypomethylation (logFC < 0) relative to U2OS parental cells.

### Enrichment bias calculation and hypergeometric distribution testing

Enrichment bias calculations were done by first determining the following values for each feature (e.g., genomic annotation, replication timing):

*q* = Number of CpGs that are differentially methylated in feature (e.g., exon)

*m* = Total number of CpGs on the EPIC array that match feature (e.g., exon)

*n* = Total number CpGs on the EPIC array that do not match feature (e.g., everything that is not an exon)

*k* = Total number of all differentially methylated CpGs

Next, the expected number of CpGs that would be differentially methylated in that feature by random chance was determined with the following equation:

$$e=\left(\frac{m}{m+n}\right)k$$

Finally, percentage enrichment bias was calculated with the following equation:

$$\%\;enrichment\;bias=\left(\frac{q-e}{k}\right)\times 100,$$

where positive or negative enrichment values indicate more or less enrichment for a feature than would be expected by random chance, respectively.

Hypergeometric distribution testing for determining significance of enrichment bias was performed using the phyper() function in R with the following values: *q*, *m*, *n*, *k*.

### Data access

EPIC array data can be found under GEO Accession # GSE137913.

To review GEO accession GSE137913:

Go to https://www.ncbi.nlm.nih.gov/geo/query/acc.cgi?acc=GSE137913

### Signature evaluation in TCGA BRCA samples

Upper quartile normalized RSEM gene expression data for TCGA BRCA (*n* = 1201) was downloaded from the GDC legacy archive (https://portal.gdc.cancer.gov). The data was log2 transformed and median centered. To determine the per sample UB signature score, the samples were ranked by the median expression of the 145 UB gene signature. Samples were then divided at the median and grouped as high or low based on rank. Copy number burden, aneuploidy, and homologous recombination deficiency data were extracted from Thorsoon and colleagues [100] and plotted by UB signature group and PAM50 subtype [101]. Significance was calculated by *t* test. The CIN70 score was determined as previously described in Fan and colleagues [102]. The CIN70 was plotted against the UB, colored by PAM50 subtype, and r^2^ and Pearson correlation were calculated. All analysis was performed in R (v3.5.2).

### Cdh1 pulldown for analysis of interactors by mass spectrometry

FLAG-tagged Cdh1 was expressed in HEK-293T cells for 24 h by transient transfection. Transfections were performed on 150 mm dishes (8 per condition) using Mirus TransIT-LT1 Transfection Reagent (Mirus Bio, Madison, Wisconsin) and Lipofectamine 2000 (Life Technologies). Cells were treated with MG-132 (10 μM for 4 h) in culture prior to lysis, dislodged by trypsinization, washed with PBS, and lysed in NETN supplemented with 2 μg/mL pepstatin, 2 μg/mL apoprotinin, 10 μg/mL leupeptin, 1 mM AEBSF, 1 mM Na_3_VO_4_, and 1 mM NaF on ice for 20 min. Cell lysates were then clarified by centrifugation at 15,000 rpm for 15 min.

Anti-FLAG M2 agarose (Sigma, catalog no. F2426) was used for precipitation (6 h at 4°C). The beads were washed with NETN 3 times and eluted twice with 150 μL of 0.1 M Glycine-HCl (pH 2.3) and then neutralized with Tris 1M (pH 10.0). The total eluted protein was reduced (5 mM DTT) and alkylated using iodoacetamide (1.25 mM) for 30 min in the dark. The resultant protein was then digested overnight with sequencing grade trypsin (Promega). The trypsin:protein ratio was maintained at 1:100. Total peptides were purified on Pierce C18 spin columns (Cat 89870) using the manufacturer’s protocol. Peptides were eluted using 70% acetonitrile and 0.1% TFA solution in 50 μL volumes twice, dried on a SpeedVac at room temperature, and processed by mass spectrometry proteomic analysis.

### Mass spectrometry

Peptides were separated by reversed-phase nano-high-performance liquid chromatography using a nanoAquity UPLC system (Waters Corp., Milford, Massachusetts). Peptides were first trapped in a 2-cm trapping column (Acclaim PepMap 100, C18 beads of 3.0 μm particle size, 100 Å pore size) and a 25-cm EASY-spray analytical column (75 μm inner diameter, C18 beads of 2.0 μm particle size, 100 Å pore size) at 35°C. The flow rate was 250 nL/minute over a gradient of 1% buffer B (0.1% formic acid in acetonitrile) to 30% buffer B in 150 min, and an in-line Orbitrap Elite mass spectrometer (Thermo Scientific) performed mass spectral analysis. The ion source was operated at 2.6 kV with the ion transfer tube temperature set at 300°C. A full MS scan (300 to 2000 m/z) was acquired in Orbitrap with a 120,000 resolution setting, and data-dependent MS2 spectra were acquired in the linear ion trap by collision-induced dissociation using a 2.0 m/z wide isolation window on the 15 most intense ions. Precursor ions were selected based on charge states (+2, +3) and intensity thresholds (above 1e5) from the full scan; dynamic exclusion (one repeat during 30 s, a 60-s exclusion time window) was also used. The polysiloxane lock mass of 445.120030 was used throughout spectral acquisition.

Raw mass spectrometry data files were searched using SorcererTM-SEQUEST (build 5.0.1, Sage N Research), the Transproteomic Pipeline (TPP v4.7.1), and Scaffold (v4.4.1.1) with the UniProtKB/Swiss-Prot human canonical sequence database (20,263 entries; release 07/2013). The search parameters used were a precursor mass between 400 and 4500 amu, zero missed cleavages, a precursor ion tolerance of 3 amu, accurate mass binning within PeptideProphet, fully tryptic digestion, a static carbamidomethyl cysteine modification (+57.021465), variable methionine oxidation (+15.99492), and variable serine, threonine and tyrosine (STY) phosphorylation (79.966331). A 1% protein-level FDR was determined by Scaffold.

## Supporting information

S1 Fig Analysis of putative APC/C substrates. (A) U2OS cells were arrested in mitosis with nocodazole, collected by shake-off, treated with the CDK1 inhibitor RO-3306, and harvested for immunoblot at the indicated time points. Cyclin B and NUSAP1 serve as positive APC/C controls. Data representative of *n* = 3 experiments. (B) U2OS cells were transiently transfected with the indicated plasmids, arrested in mitosis with nocodazole, collected by shake-off, treated with the CDK1 inhibitor RO-3306, and harvested for immunoblot after 2 h. FoxM1 serves as a positive control for APC/C activation. Data representative of *n* = 3 experiments. (C) HeLa and U2OS cells were synchronized in mitosis by nocodazole and released by mitotic shake-off. Time points were collected as shown and analyzed by immunoblot. FoxM1 serves as positive APC/C control that is degraded at M/G1 phases. Data representative of *n* = 2 experiments.
(TIF)

S2 Fig TTF2 is ubiquitylated by APC/C in vitro. (A) Ubiquitylation reactions of TTF2* by UBE2C using methylated Ub or wild-type Ub (lanes 1–6) in combination with APC/C^Cdh1^, APC/C alone, or Cdh1 alone. Ubiquitylation reactions of TTF2* by both E2s, UBE2C, and UBE2S, (lanes 7–9) in combination with APC/C^Cdh1^, APC/C alone, or Cdh1 alone. Ubiquitylation was detected by fluorescence scanning at 60 min time points. Data representative of *n* = 3 experiments. (B) Ubiquitylation reactions with APC/C^Cdh1^, UBE2C, FL NASP*, or control CyclinB*, and wild-type ubiquitin. NASP* and CyclinB* were detected by fluorescence scanning (* indicates fluorescently labeled protein). Data representative of *n* = 2 experiments.
(TIF)

S3 Fig UHRF1 protein levels are cell cycle regulated and sensitive to APC/C inhibition with the small-molecule inhibitor proTAME. (A) HeLa cells were synchronized in mitosis, collected by shake-off, released into the cell cycle, and analyzed by immunoblot at the indicated time points. Data representative of *n* = 3 experiment. (B) U2OS cells were synchronized in mitosis, collected by shake-off, released into the cell cycle, and analyzed by immunoblot at the indicated time points. Line indicates samples that were run on separate gels, with appropriate corresponding loading controls for each gel. Data representative of *n* = 3 experiments. (C) HCT116 and U2OS cells were released into G1 from a mitotic block for 1.5 h and then were subsequently treated with proTAME for 1.5 h. Endogenous UHRF1 and Cdh1 were analyzed by immunoblot. Data representative of *n* = 1 experiment.
(TIF)

S4 Fig UHRF1 ubiquitylation by APC/C. (A) Ubiquitylation reactions of FL-UHRF1* by UBE2C with either methylated Ub or wild-type Ub. Reactions were performed using UHRF1^WT^ or a variant harboring alanine substitution in the KEN-box (KEN:AAA). KEN degron motif mutants in UHRF1 are shown in lanes 4 and 8. Ubiquitylation was detected by fluorescence scanning at 30 min time points. Data representative of *n* = 3 experiments. (B) Ubiquitylation reactions of LPS-UHRF1* by UBE2C with either methylated Ub or wild-type Ub. Reactions were performed using UHRF1^WT^ or a variant harboring alanine substitution in the KEN-box (KEN:AAA). KEN degron motif mutants in UHRF1 are shown in lanes 4 and 8. Ubiquitylation was detected by fluorescence scanning at 30 min time points. Data representative of *n* = 3 experiments. (C) Ubiquitylation reactions of FL-UHRF1* and LPS-UHRF1* are exclusive to Cdh1 as the coactivator. Ubiquitylation reactions were performed using wild-type APC/C^Cdh1^, which can only utilize Cdh1, but not Cdc20, as well as pE-APC/C^Cdh1^, which mimics the APC/C phosphorylated state and can therefore use either Cdc20 or Cdh1. In parallel, we analyzed ubiquitylation of CycB^NTD*^ and Securin*, which can be ubiquitylated by both APC/C^Cdc20^ and APC/C^Cdh1^. Data representative of *n* = 3 experiments.
(TIF)

S5 Fig UHRF1 depletion impairs chromosome alignment. (A) U2OS cells were treated with control siRNAs targeting firefly luciferase or three independent UHRF1 siRNAs. After 48 h, cells were treated with EdU, harvested 30 min later, and analyzed by flow cytometry for EdU incorporation and DNA content. Flow cytometry blots are shown (top) and quantification of the percent of cells in each cell cycle phase (bottom). Data representative of 3 independent experiments, each analyzing >10,000 cells per condition. (S1 Data) (B) HCT116 cells were depleted of UHRF1 using 2 independent siRNA oligonucleotides. Cells were fixed and stained with antibodies to the kinetochore protein CENP-C and microtubules. Data representative of *n* = 2 experiments, counting a total of 319 mitotic cells (control), 318 mitotic cells (siUHRF1-1), and 329 mitotic cells (siUHRF1-2) (these numbers are the sum of 2 replicates). (S1 Data)
(TIF)

S6 Fig Progression through S/G2 phases in cells expressing nondegradable UHRF1. (A) HeLa S3 cells stably expressing GFP-UHRF1^WT^ or GFP-UHRF1^KEN:AAA^ were synchronized at G1/S by double thymidine block, released in the cell cycle, and analyzed by immunoblot at the indicated time points. Cells progressed through S/G2 phases with minimal differences except for an increase in cyclin E levels. Data representative of *n* = 1 experiment. (B) Asynchronous RPE-1 cells stably expressing GFP-UHRF1^WT^ or GFP-UHRF1^KEN:AAA^ were harvested for immunoblotting for cell cycle markers as shown. Data representative of *n* = 1 experiment. (C) Asynchronous HeLa S3 cells stably expressing GFP-UHRF1^WT^ or GFP-UHRF1^KEN:AAA^ along with 3'UTR targeting shUHRF1 were harvested for immunoblotting for cell cycle markers as shown. Data representative of *n* = 1 experiment.
(TIF)

S7 Fig A 145 gene signature derived from KEN-containing proteins, which have cell cycle–dependent gene transcription, is associated with makers of chromosome instability in breast cancer. (A) TCGA BRCA samples (*n* = 1,201) were assigned to High or Low based on the ranked median value of the 145 gene signature score. Samples were then plotted for the given genomic feature based on Thorsson and colleagues by both gene signature group and PAM50 subtype. Significant was determined by *t* test or ANOVA where appropriate. The median 145 gene signature score was plotted against the chromosome instability score (CIN70) (r2 = 0.72, Pearson correlation *p* < 0.001). Colors indicate PAM50 subtypes. (S1 Data)
(TIF)

S1 Data The underlying raw data for all relevant figures. (XLSX)

S2 Data FLAG:Cdh1 IP from 293T cells, analyzed by mass spectrometry. (XLSX)

S3 Data. Data tables protein and gene information for (1) human proteins containing a KEN-sequence motifs; (2) genes that are cell cycle regulated based on previous transcriptomic studies; and (3) the overlapping set of genes/proteins which are putative APC/C substrates (XLSX)

S4 Data Comparison of APC/C substrates identified here to other studies which identified APC/C substrates using alternative methods. (XLSX)

S5 Data Proteins tested as potential APC/C substrates in this study using different methods. (XLSX)

S6 Data List of siRNA, shRNA, plasmids, primers, and antibodies and their respective sources used in this study. (XLSX)

S1 Text Code used in R to analyze EPIC array data. (TXT)

## Abbreviations

APC/C: anaphase-promoting complex/cyclosome
coIP: co-immunoprecipitation
DMEM: Dulbecco’s Modified Eagle Medium
FBS: fetal bovine serum
FL: full-length
GO: gene ontology
GPS: Global Protein Stability Profiling
IP: immunoprecipitation
MDS: multidimensional scaling
STY: serine, threonine and tyrosine
TTS: transcription termination sites
UHRF1: ubiquitin-like with PHD and RING finger domains 1
Ub: ubiquitin
WB: western blotting
GEO: Gene Expression Omnibus
