# Supplementary figures and images for: In silico APC/C substrate discovery reveals cell cycle-dependent degradation of UHRF1 and other chromatin regulators
Source: PLoS Biol. 2020 Dec 11;18(12):e3000975. doi: 10.1371/journal.pbio.3000975 (PMC7758050; doi:10.1371/journal.pbio.3000975)

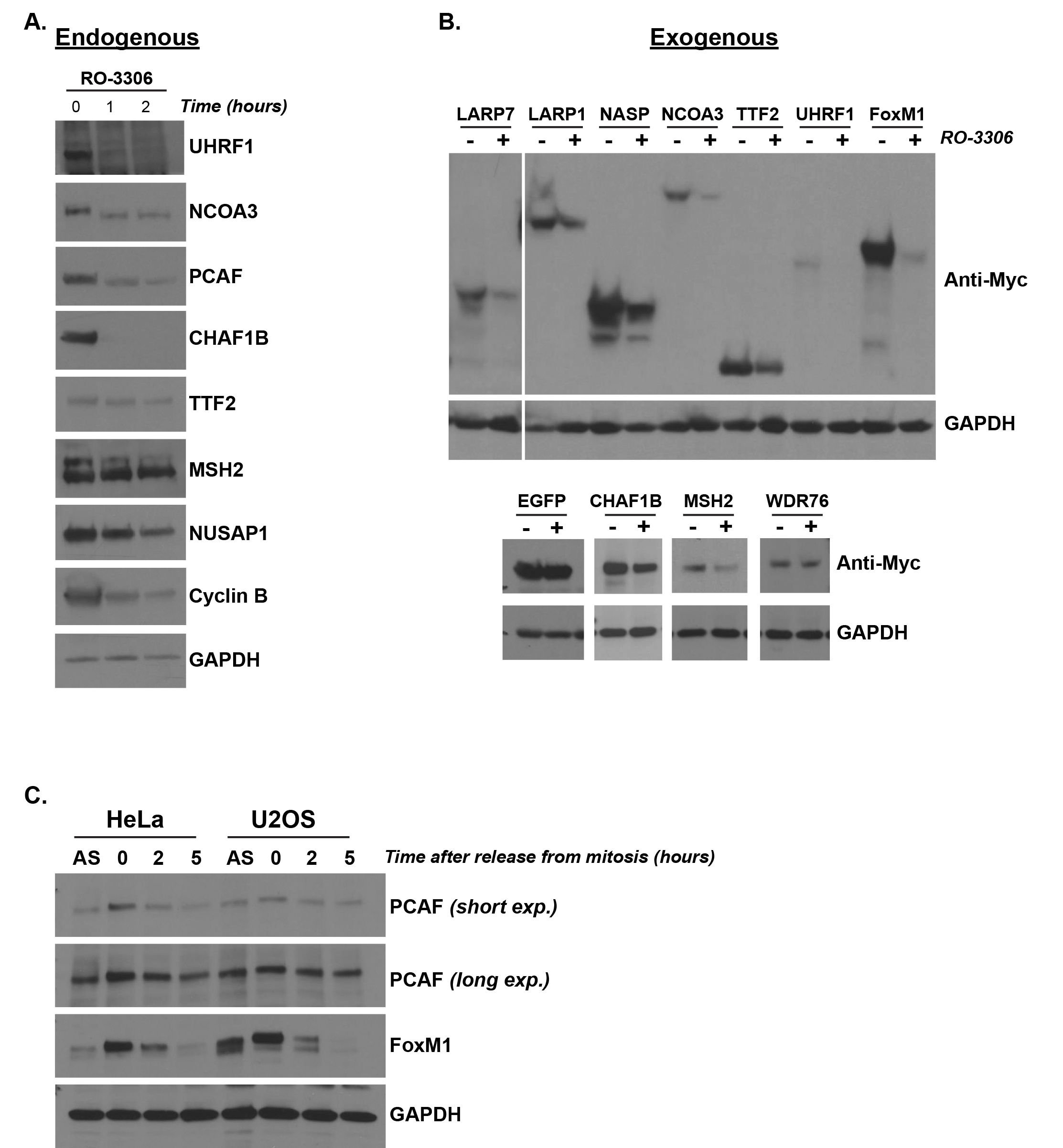

Supplement: S1 Fig — (A) U2OS cells were arrested in mitosis with nocodazole, collected by shake-off, treated with the CDK1 inhibitor RO-3306, and harvested for immunoblot at the indicated time points. Cyclin B and NUSAP1 serve as positive APC/C controls. Data representative of n = 3 experiments. (B) U2OS cells were transiently transfected with the indicated plasmids, arrested in mitosis with nocodazole, collected by shake-off, treated with the CDK1 inhibitor RO-3306, and harvested for immunoblot after 2 h. FoxM1 serves as a positive control for APC/C activation. Data representative of n = 3 experiments. (C) HeLa and U2OS cells were synchronized in mitosis by nocodazole and released by mitotic shake-off. Time points were collected as shown and analyzed by immunoblot. FoxM1 serves as positive APC/C control that is degraded at M/G1 phases. Data representative of n = 2 experiments. (TIF) [file pbio.3000975.s001.tif]

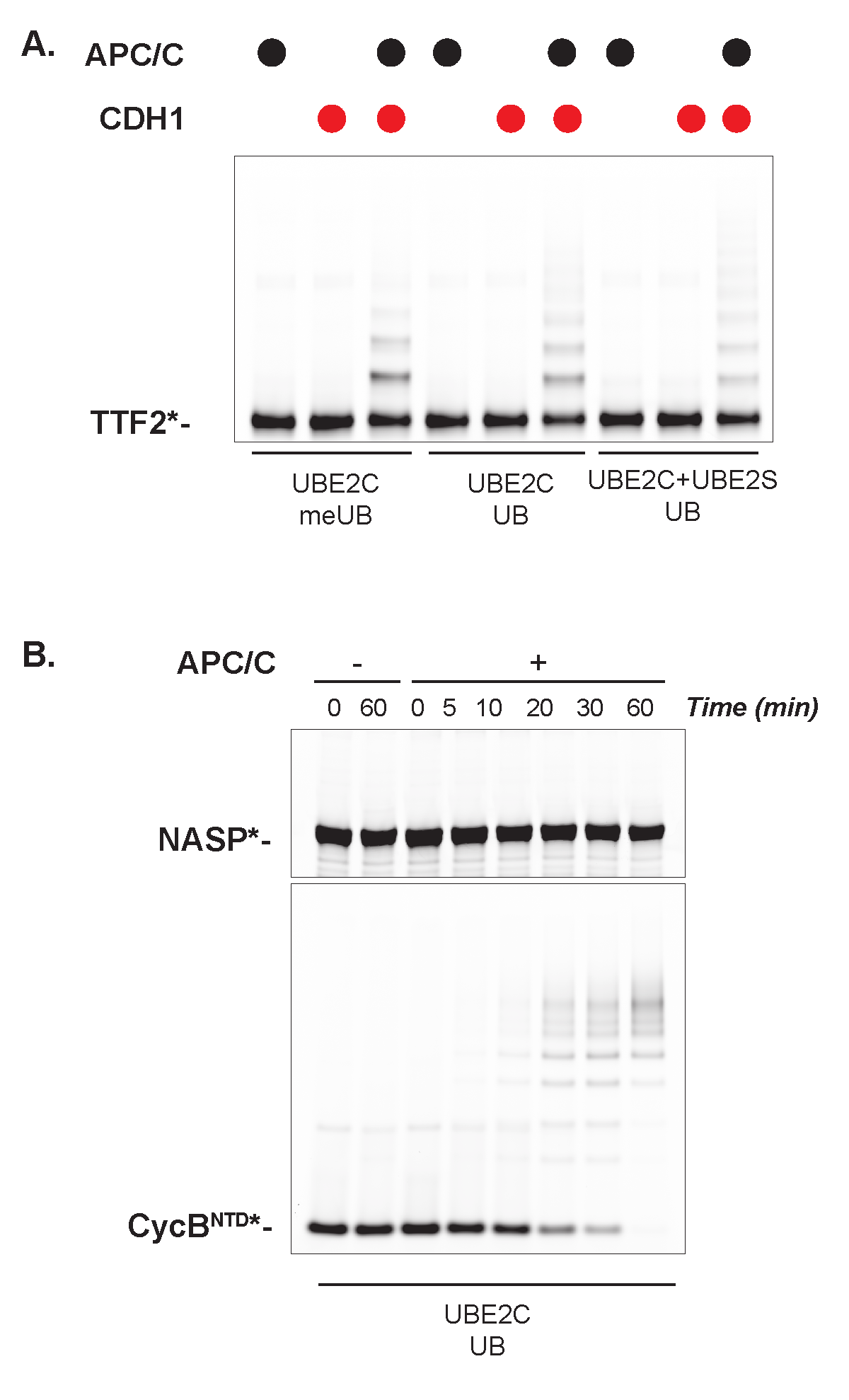

Supplement: S2 Fig — (A) Ubiquitylation reactions of TTF2* by UBE2C using methylated Ub or wild-type Ub (lanes 1–6) in combination with APC/CCdh1, APC/C alone, or Cdh1 alone. Ubiquitylation reactions of TTF2* by both E2s, UBE2C, and UBE2S, (lanes 7–9) in combination with APC/CCdh1, APC/C alone, or Cdh1 alone. Ubiquitylation was detected by fluorescence scanning at 60 min time points. Data representative of n = 3 experiments. (B) Ubiquitylation reactions with APC/CCdh1, UBE2C, FL NASP*, or control CyclinB*, and wild-type ubiquitin. NASP* and CyclinB* were detected by fluorescence scanning (* indicates fluorescently labeled protein). Data representative of n = 2 experiments. (TIF) [file pbio.3000975.s002.tif]

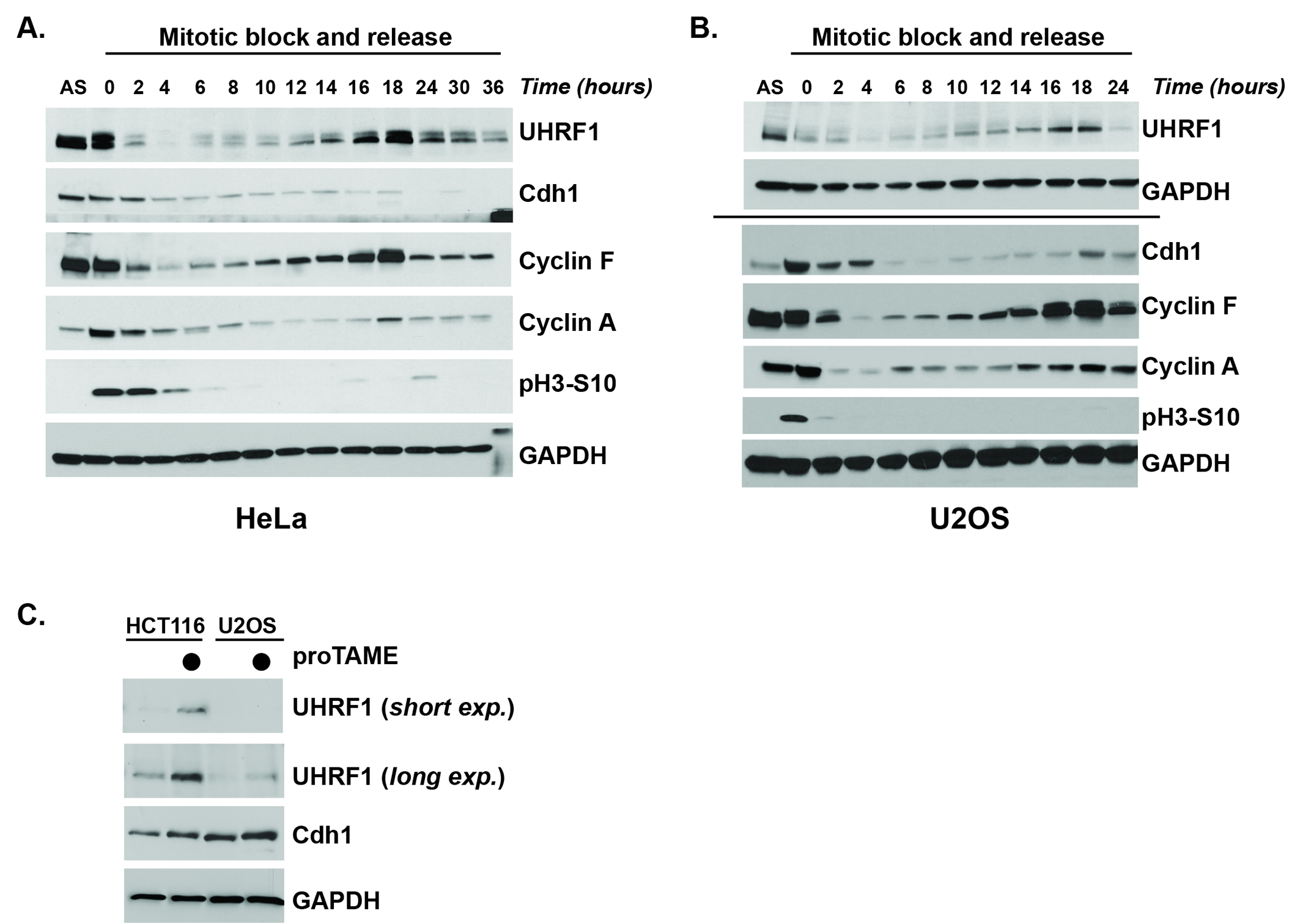

Supplement: S3 Fig — (A) HeLa cells were synchronized in mitosis, collected by shake-off, released into the cell cycle, and analyzed by immunoblot at the indicated time points. Data representative of n = 3 experiment. (B) U2OS cells were synchronized in mitosis, collected by shake-off, released into the cell cycle, and analyzed by immunoblot at the indicated time points. Line indicates samples that were run on separate gels, with appropriate corresponding loading controls for each gel. Data representative of n = 3 experiments. (C) HCT116 and U2OS cells were released into G1 from a mitotic block for 1.5 h and then were subsequently treated with proTAME for 1.5 h. Endogenous UHRF1 and Cdh1 were analyzed by immunoblot. Data representative of n = 1 experiment. (TIF) [file pbio.3000975.s003.tif]

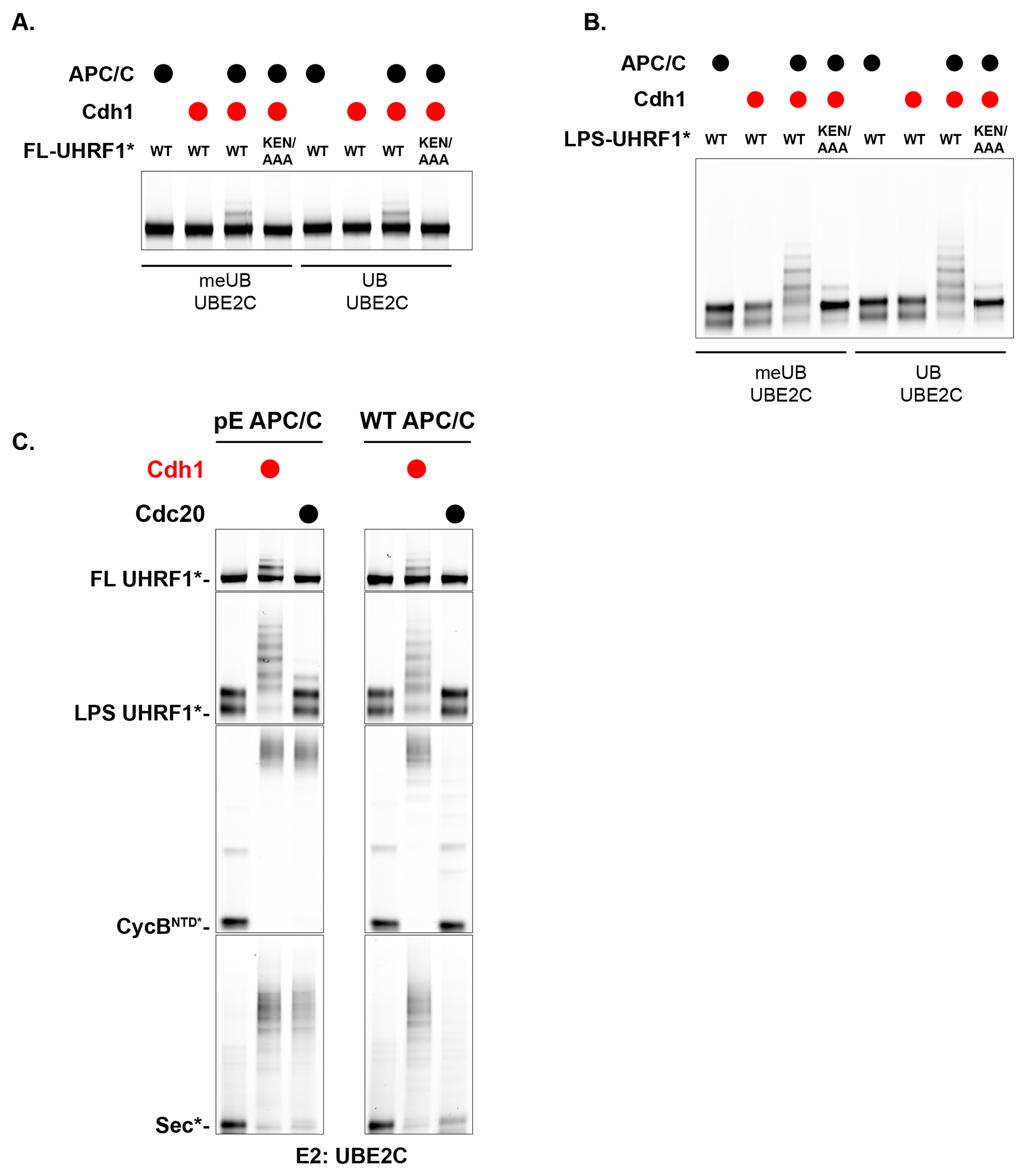

Supplement: S4 Fig — (A) Ubiquitylation reactions of FL-UHRF1* by UBE2C with either methylated Ub or wild-type Ub. Reactions were performed using UHRF1WT or a variant harboring alanine substitution in the KEN-box (KEN:AAA). KEN degron motif mutants in UHRF1 are shown in lanes 4 and 8. Ubiquitylation was detected by fluorescence scanning at 30 min time points. Data representative of n = 3 experiments. (B) Ubiquitylation reactions of LPS-UHRF1* by UBE2C with either methylated Ub or wild-type Ub. Reactions were performed using UHRF1WT or a variant harboring alanine substitution in the KEN-box (KEN:AAA). KEN degron motif mutants in UHRF1 are shown in lanes 4 and 8. Ubiquitylation was detected by fluorescence scanning at 30 min time points. Data representative of n = 3 experiments. (C) Ubiquitylation reactions of FL-UHRF1* and LPS-UHRF1* are exclusive to Cdh1 as the coactivator. Ubiquitylation reactions were performed using wild-type APC/CCdh1, which can only utilize Cdh1, but not Cdc20, as well as pE-APC/CCdh1, which mimics the APC/C phosphorylated state and can therefore use either Cdc20 or Cdh1. In parallel, we analyzed ubiquitylation of CycBNTD* and Securin*, which can be ubiquitylated by both APC/CCdc20 and APC/CCdh1. Data representative of n = 3 experiments. (TIF) [file pbio.3000975.s004.tif]

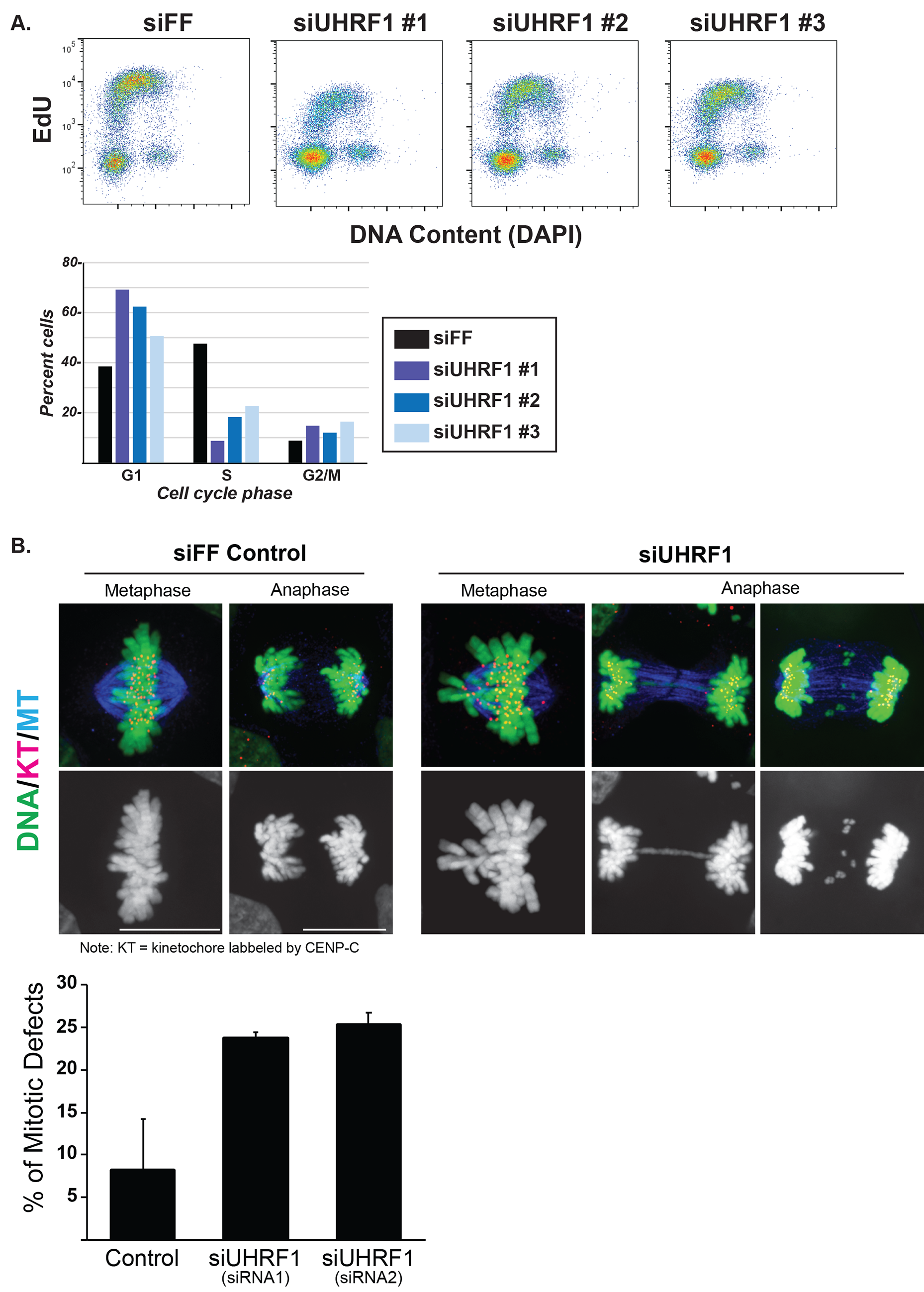

Supplement: S5 Fig — (A) U2OS cells were treated with control siRNAs targeting firefly luciferase or three independent UHRF1 siRNAs. After 48 h, cells were treated with EdU, harvested 30 min later, and analyzed by flow cytometry for EdU incorporation and DNA content. Flow cytometry blots are shown (top) and quantification of the percent of cells in each cell cycle phase (bottom). Data representative of 3 independent experiments, each analyzing >10,000 cells per condition. (S1 Data) (B) HCT116 cells were depleted of UHRF1 using 2 independent siRNA oligonucleotides. Cells were fixed and stained with antibodies to the kinetochore protein CENP-C and microtubules. Data representative of n = 2 experiments, counting a total of 319 mitotic cells (control), 318 mitotic cells (siUHRF1-1), and 329 mitotic cells (siUHRF1-2) (these numbers are the sum of 2 replicates). (S1 Data) (TIF) [file pbio.3000975.s005.tif]

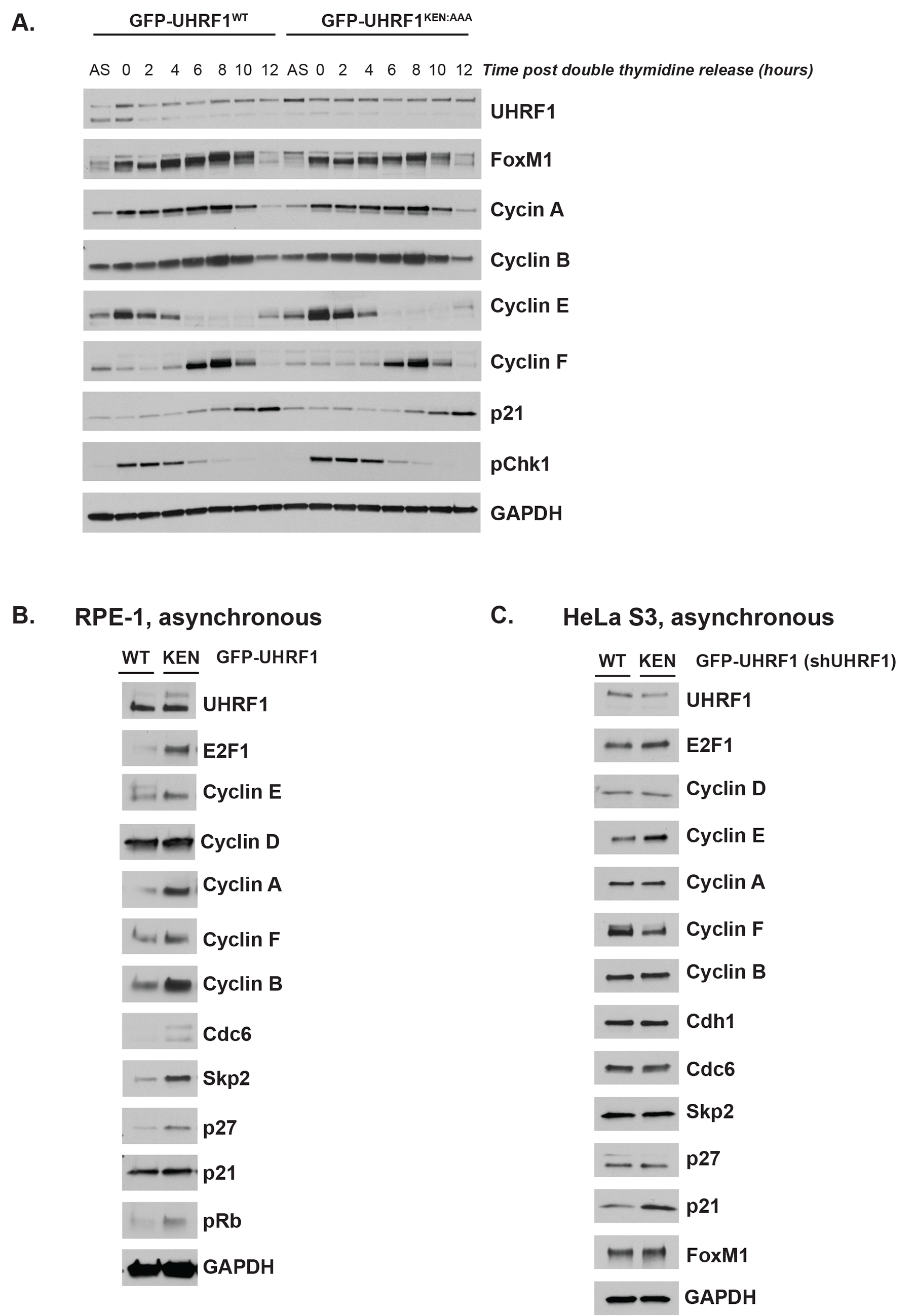

Supplement: S6 Fig — (A) HeLa S3 cells stably expressing GFP-UHRF1WT or GFP-UHRF1KEN:AAA were synchronized at G1/S by double thymidine block, released in the cell cycle, and analyzed by immunoblot at the indicated time points. Cells progressed through S/G2 phases with minimal differences except for an increase in cyclin E levels. Data representative of n = 1 experiment. (B) Asynchronous RPE-1 cells stably expressing GFP-UHRF1WT or GFP-UHRF1KEN:AAA were harvested for immunoblotting for cell cycle markers as shown. Data representative of n = 1 experiment. (C) Asynchronous HeLa S3 cells stably expressing GFP-UHRF1WT or GFP-UHRF1KEN:AAA along with 3'UTR targeting shUHRF1 were harvested for immunoblotting for cell cycle markers as shown. Data representative of n = 1 experiment. (TIF) [file pbio.3000975.s006.tif]

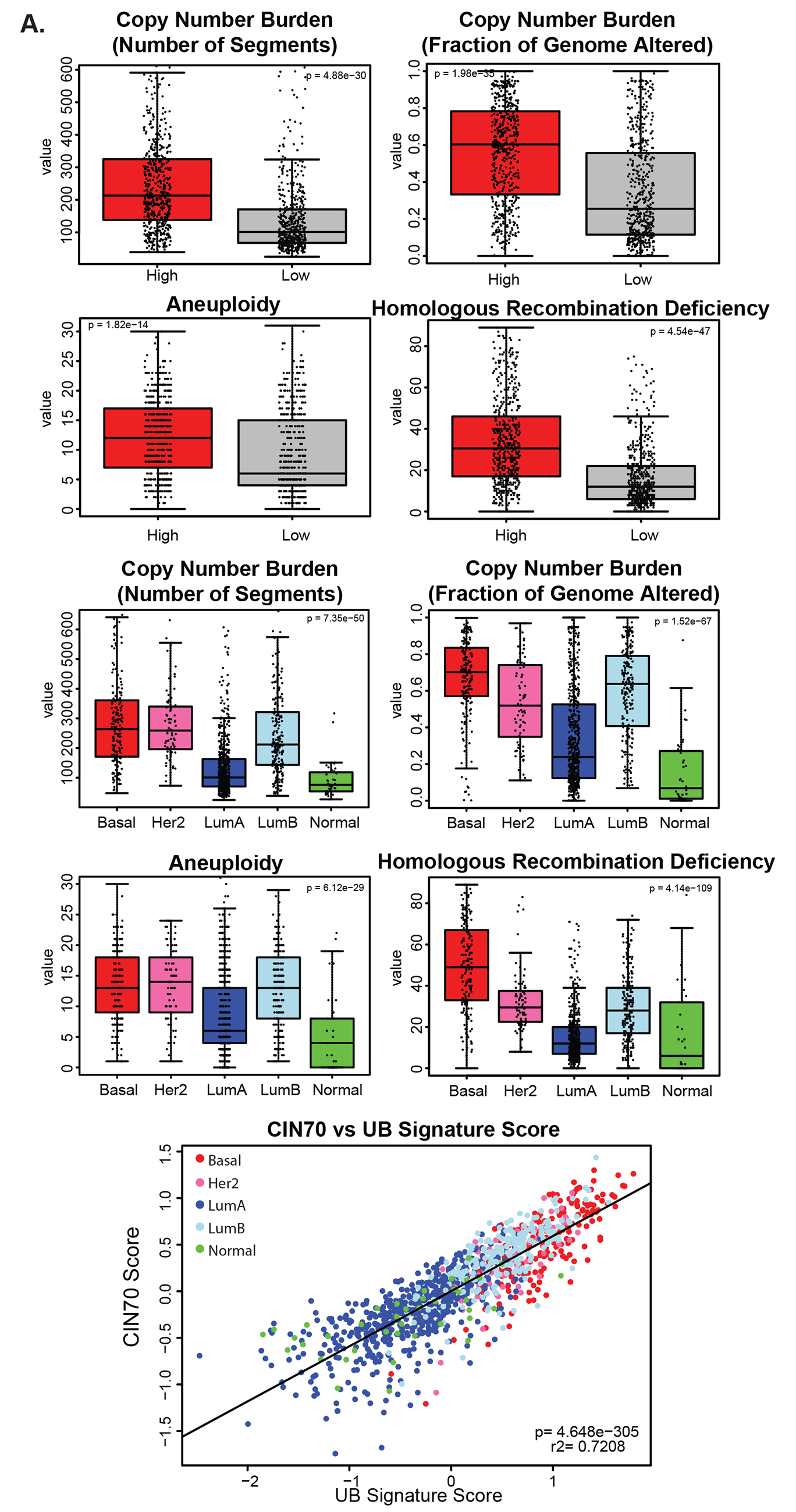

Supplement: S7 Fig — (A) TCGA BRCA samples (n = 1,201) were assigned to High or Low based on the ranked median value of the 145 gene signature score. Samples were then plotted for the given genomic feature based on Thorsson and colleagues by both gene signature group and PAM50 subtype. Significant was determined by t test or ANOVA where appropriate. The median 145 gene signature score was plotted against the chromosome instability score (CIN70) (r2 = 0.72, Pearson correlation p < 0.001). Colors indicate PAM50 subtypes. (S1 Data) (TIF) [file pbio.3000975.s007.tif]
